# From Unprintable Peptidic Gel to Unstoppable: Transforming Diphenylalanine Peptide (Fmoc-FF) Nanowires and Cellulose Nanofibrils into a High-Performance Biobased Gel for 3D Printing

**DOI:** 10.1021/acsabm.4c01803

**Published:** 2025-03-07

**Authors:** Feras Dalloul, J. Benedikt Mietner, Dhanya Raveendran, Shouzheng Chen, Enguerrand Barba, Dennis M. J. Möck, Fabio Hubel, Benedikt Sochor, Sarathlal Koyiloth Vayalil, Linnea Hesse, Andrea Olbrich, Jörn Appelt, Peter Müller-Buschbaum, Stephan V. Roth, Julien R. G. Navarro

**Affiliations:** † Institute of Wood Science, Universität Hamburg, Haidkrugsweg 1, 22885 Barsbüttel, Germany; ‡ Federal Research Institute for Rural Areas, Forestry and Fisheries, Institute of Wood Research, Johann Heinrich von Thünen Institute, Haidkrugsweg 1, 22885 Barsbüttel, Germany; § Deutsches Elektronen-Synchrotron DESY, Notkestrasse 85, 22607 Hamburg, Germany; ∥ Advanced Light Source, Lawrence Berkeley National Laboratory, 6 Cyclotron Road, Berkeley, California 94720, United States; ⊥ Applied Science Cluster, UPES, Dehradun, Uttarakhand 248007, India; # Department of Physics, Chair for Functional Materials, TUM School of Natural Sciences, Technical University of Munich, James-Franck-Strasse 1, 85748 Garching, Germany; ∇ Department of Fibre and Polymer Technology, KTH Royal Institute of Technology, Teknikringen 56-58, 10044 Stockholm, Sweden

**Keywords:** cellulose nanofibrils (CNF), single electron transfer living radical polymerization (SET-LRP), 3D gel printing, direct ink writing (DIW), nanocellulose, Fmoc-FF

## Abstract

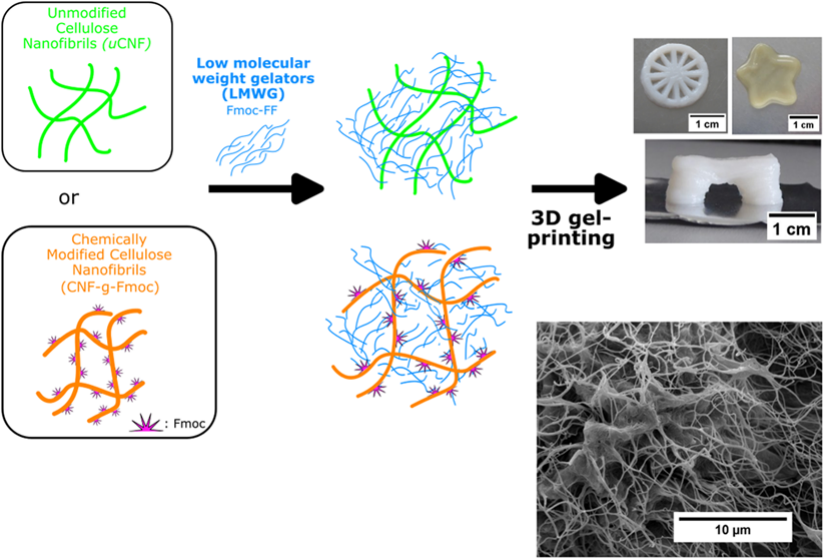

The growing interest in gel-based additive manufacturing, also known as three-dimensional (3D) gel-printing technology, for research underscores the crucial need to develop robust biobased materials with excellent printing quality and reproducibility. The main focus of this study is to prepare and characterize some composite gels obtained with a low-molecular-weight gelling (LMWG) peptide called Fmoc-diphenylalanine (Fmoc-FF) and two types of cellulose nanofibrils (CNFs). The so-called Fmoc-FF peptide has the ability to self-assemble into a nanowire shape and therefore create an organized network that induces the formation of a gel. Despite their ease of preparation and potential use in biological systems, unfortunately, those Fmoc-FF nanowire gel systems cannot be 3D printed due to the high stiffness of the gel. For this reason, this study focuses on composite materials made of cellulose nanofibrils and Fmoc-FF nanowires, with the main objective being that the composite gels will be suitable for 3D printing applications. Two types of cellulose nanofibrils are employed in this study: (1) unmodified pristine cellulose nanofibrils (uCNF) and (2) chemically modified cellulose nanofibrils, which ones have been grafted with polymers containing the Fmoc unit on their backbone (CNF-*g*-Fmoc). The obtained products were characterized through solid-state cross-polarization magic angle-spinning ^1^H NMR and confocal laser scanning microscopy. Within these two CNF structures, two composite gels were produced: uCNF/Fmoc-FF and CNF-*g*-Fmoc/Fmoc-FF. The mechanical properties and printability of the composites are assessed using rheology and challenging 3D object printing. With the addition of water, different properties of the gels were observed. In this instance, CNF-*g*-Fmoc/Fmoc-FF (*c* = 5.1%) was selected as the most suitable option within this product range. For the composite bearing uCNF, exceptional print quality and mechanical properties are achieved with the CNF/Fmoc-FF gel (*c* = 5.1%). The structures are characterized by using field emission scanning electron microscopy (FESEM) and small-angle X-ray scattering (SAXS) measurements.

## Introduction

Three-dimensional (3D) printing or additive manufacturing, has made significant advances in a wide range of industries through its ability to provide flexible design features, customized object production, rapid manufacturing processes, and precise control over structural defects and details.^1−3^ 3D gel-printing, or direct ink writing (DIW), has recently raised the interest of the scientific community as it can 3D-print (hydro)gels at ambient temperature, making it possible to create complex and functional gel structures with unprecedented precision and customization.^4−6^ Various materials are available for 3D bioprinting, including hydrogels,^7^ extracellular matrix (ECM),^8^ bioinks,^9^ synthetic polymers,^10^ and composites.^11^ The choice of the starting gel materials, the ink, depends on the final application and the targeted template, with emphasis on biocompatibility and cell viability to enable possible tissue regeneration.^12^ Biopolymers, such as alginates or collagens, provide a favorable environment for cell growth but often lack necessary mechanical properties (low Young’s modulus and low mechanical strength). However, the addition of a cross-linking agent, or a ultraviolet (UV) curing process, can overcome this limitation.^13−15^ Carefully calibrated print settings combined with an appropriate gel can be used to achieve a consistent 3D structure. The gel should, hereby, ideally require minimal processing and provide impressive stability.

Low-molecular-weight gelators (LMWGs) have attracted considerable interest due to their ability to self-assemble and form gel networks, with potential applications in areas such as drug delivery, tissue engineering, and sensor development.^16^ Fluorenyl-9-methoxycarbonyl (Fmoc)-diphenylalanine (Fmoc-FF), composed of two phenylalanine (FF) amino acids protected by a fluorenylmethyloxycarbonyl (Fmoc) group, is a widely known and studied peptide that has demonstrated excellent gelation properties, ease of preparation, low toxicity, and potential for use in biological systems.^17^ There are two pathways for triggering the gelation process: solvent switching and pH-induced.^18^ In the latter, gelation occurs by lowering the pH of the predissolved Fmoc-FF solution from pH 10 to 4, where Fmoc-FF molecules undergo pH-dependent self-assembly into a gel.^19^ Solvent switching is a technique that involves the dissolution of Fmoc-FF in a suitable organic solvent (dimethyl sulfoxide (DMSO), ethanol) followed by the addition of water, which reduces its solubility. This approach allows the sequential dissolution of the Fmoc-FF immediately before controlled aggregation processes, thus simplifying and speeding up the process of self-assembly. Zhao et al.^20^ proposed a mechanism whereby Fmoc-FF self-assembles into a nanocylindrical structure (∼3.0 nm diameter) through interlocking π–π interactions of four twisted antiparallel β-sheets.

3D printing of low-molecular-weight gels (LMWGs) has not been extensively documented, and developing these processing techniques with LMWGs may offer significant potential for complex hierarchical tissue engineering applications. Solvent-triggered Fmoc-FF gels with spherulitic domains composed of densely packed fibers can be 3D printed, as demonstrated by Nolan et al.^21^ However, due to the stiffness of the gel, 3D printing of pure Fmoc-FF gels remains challenging.

The microstructure of the formed gels can be influenced by the method of gelation, and the desired sphere-like microstructure can be produced with different LMWGs and solvents. Despite exhibiting good mechanical properties, Fmoc-FF hydrogels are inherently rigid, lacking elasticity resulting in fragmentation during extrusion, thus rendering them difficult to process by 3D printing.^22^ Gong et al.^23^ have succeeded in developing a new class of composite gel containing Fmoc-FF and sodium alginate (SA) with enhanced characteristics. Fmoc-FF’s self-assembly was found to be hindered by SA hydrogen and aromatic bonding and, therefore, it regulated the assembly of Fmoc-FF in the scaffold. This resulted in improved mechanical properties and elasticity of the composite gel. A possible approach to render the mechanical properties of Fmoc-FF appropriate for 3D bioprinting could be the use of cellulose nanofibrils (CNF), a biopolymer that has already proved to be effective in bioprinting.^24−27^ The use of CNF in composite systems offers numerous advantages. With its biocompatibility, noncytotoxicity, and high aspect ratio, CNF not only improves the mechanical properties of polymer composites but can also render them compatible with biological systems.^28−32^

**Scheme 1:**
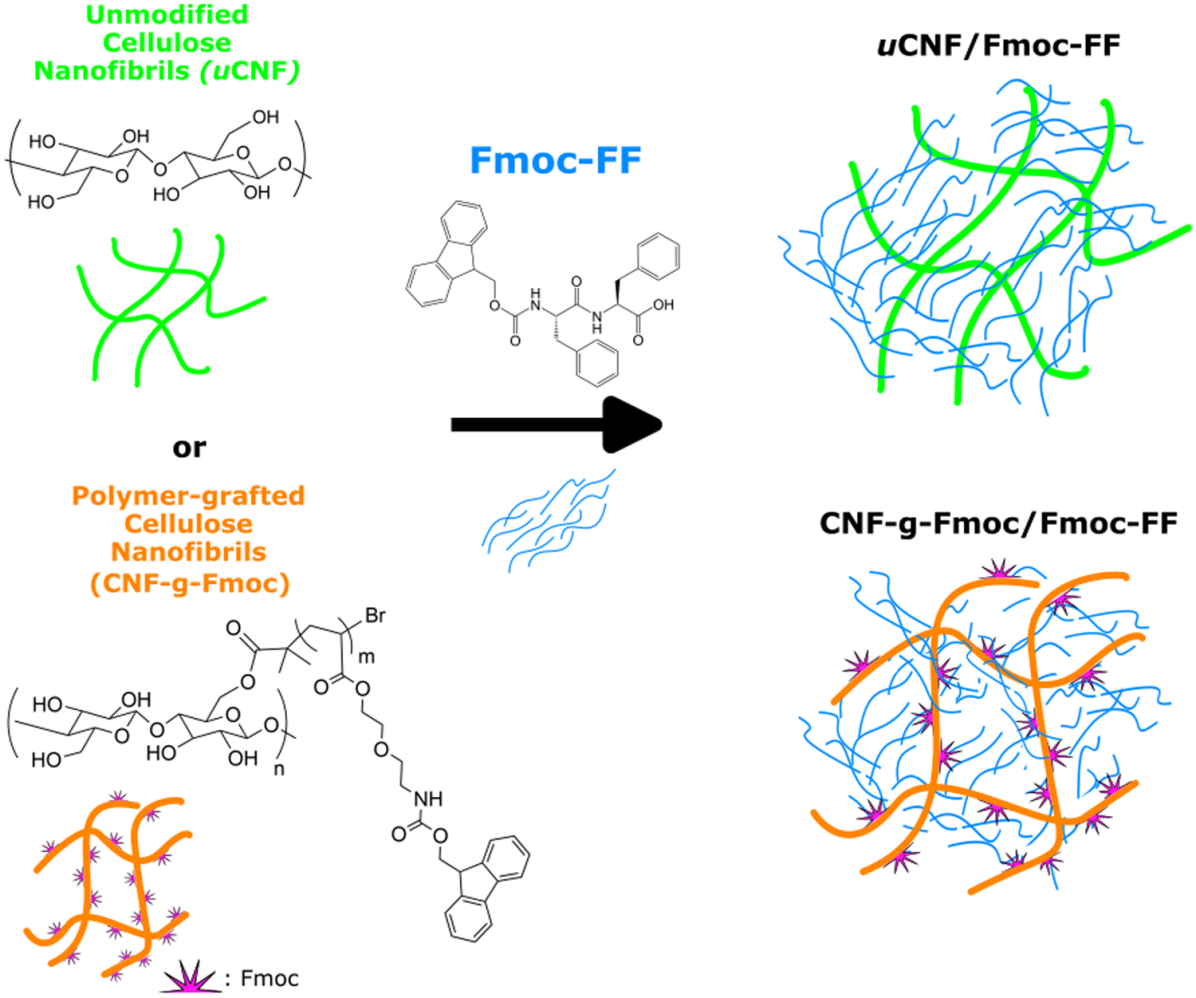
Representative Illustration of the Composition of the Two Composites uCNF/Fmoc-FF and CNF-*g*-Fmoc/Fmoc-FF

This research aims to develop a composite gel with good mechanical properties and superior 3D printing resolution. The appropriate ratio of unmodified CNF (uCNF) to Fmoc-FF is first established. Gel composites are first generated using unmodified CNF. After optimizing the gelation, and the 3D printing parameters, similar methods are used on the polymer surface-grafted CNF with Fmoc units, which is expected to facilitate π–π stacking interactions.^33,34^Scheme 1 illustrates the composition and design of the two composites. The CNF modification process involved the synthesis of a monomer containing the Fmoc function, the production of a CNF-based macroinitiator (CNF-MI), and finally the grafting of the Fmoc polymer on the CNF surface through single electron transfer living radical polymerization (SET-LRP) to yield CNF-*g*-Fmoc. In addition, the effect of the CNFs, as well as the concentration and volume fraction of DMSO as the main solvent, is studied. The gel strength is assessed through rheological studies. The chemical synthesis of the modified cellulose is analyzed using NMR spectroscopy, while the cellulose modification is examined with UV/visible (vis) spectroscopy, solid-state cross-polarization magic angle spinning (CP/MAS) ^13^C NMR, and high-resolution magic angle spinning (HR-MAS) techniques. Additionally, confocal laser scanning microscopy (CLSM), small-angle X-ray scattering (SAXS), and field emission scanning electron microscopy (FESEM) are used to analyze the morphological aspect of each sample (CNF, CNF-MI, CNF-*g*-Fmoc, uCNF/Fmoc-FF, CNF-*g*-Fmoc/Fmoc-FF). Printing is conducted using an extrusion-based 3D gel printer. The printing resolution is evaluated by printing challenging patterns.

## Materials and Methods

### Materials

1,1′-Carbonyldiimidazole (CDI, reagent grade), 2-bromo-2-methylpropionic acid 98%, imidazole >99%, sodium bicarbonate (ACS reagent, >99%), sodium chloride (ACS reagent, >99%), 2-(2-aminoethoxy) ethanol (98%), triethylamine (synthesis standard), dichloromethane (DCM), and acryloyl chloride (97%) were purchased from TCI Chemical. Dimethyl sulfoxide (DMSO, 99%) was purchased from Merck KGaA. 9-Flourenylmethoxycarbonyl chloride (Fmoc-Cl) was purchased from Carl Roth GmbH + Co. KG. Fmoc-Phe-Phe-OH (Fmoc-FF) was purchased from BLD Pharmatech (95%). Tris[2-(dimethylamino)ethyl]amine (Me_6_-TREN) was purchased from Alfa Aesar and purified by distillation (70 °C) under reduced pressure before use (colorless oil). Carbon dioxide (99.5%) was purchased from Linde AG (Pullach, Germany). Copper wire (diameter 1 mm) was purchased from Thermo Fisher Scientific. The dry cellulose source, elemental chlorine-free (ECF) bleached softwood kraft pulp, was obtained from MERCER Stendal GmbH, Germany. The Northern bleached softwood Kraft pulp was made from pine (30–60%) and spruce (40–70%).

### Fmoc-Monomer Synthesis (Fmoc-AEEA)

#### Fmoc-2-(-2-aminoethoxy) Ethanol

Sodium hydrogen carbonate (974 mg, 11.6 mmol) and Fmoc-Cl (500 mg, 1.9 mmol) were added to dry dichloromethane (DCM) (6 mL) under nitrogen. 2-(2-Aminoethoxy) ethanol (205 μL, 1.95 mmol) was then added dropwise. The reaction mixture was stirred for 16 h. The solution was then filtered, subsequently washed with distilled water and then brine, and finally dried with the addition of sodium sulfate. After solvent removal, the product (orange oil) was obtained with an 82% yield.

^1^H NMR (400 MHz, CDCl_3_) δ 7.76 (d, *J* = 7.5 Hz, 7H), 7.59 (d, *J* = 7.4 Hz, 2H), 7.40 (t, *J* = 7.4 Hz, 2H), 7.31 (t, *J* = 7.4 Hz, 2H), 5.27 (s, 1H), 4.42 (d, *J* = 6.7 Hz, 2H), 4.22 (t, *J* = 6.6 Hz, 1H), 3.72 (m, 2H), 3.55 (m, 4H), 3.40 (m, 2H), 2.22 (s, 1H).

#### (*N*-Fmoc-2-aminoethoxy)ethyl Acrylate (Fmoc-AEEA)

*N*-Fmoc-2-aminoethanol (599 mg, 1.86 mmol) was dissolved in dry DCM (20 mL) under nitrogen at 0 °C. Triethylamine (535 mL, 3.7 mmol) was then added to the solution. A solution of acryloyl chloride (270 μL, 3.4 mmol) in dry DCM (5 mL) was added dropwise to the mixture. After 2 h, the reaction was warmed to room temperature, and stirring was continued for 16 h. Finally, the reaction mixture was washed with saturated sodium bicarbonate (3×) and brine (3×) and dried with sodium sulfate. The solvent was removed to give an orange solid (yield 90%).

^1^H NMR (400 MHz, CDCl_3_) δ 7.68 (d, *J* = 7.5 Hz, 2H), 7.52 (d, *J* = 7.5 Hz, 2H), 7.32 (t, *J* = 7.5 Hz, 2H), 7.23 (td, *J* = 7.5, 1.2 Hz, 2H), 6.34 (d, *J* = 1.4 Hz, 1H), 6.08 (d, *J* = 6.9 Hz, 1H), 5.75 (dd, *J* = 10.4, 1.4 Hz, 1H), 5.13 (t, *J* = 5.9 Hz, 1H), 4.32 (d, *J* = 7.0 Hz, 2H), 4.25 (t, *J* = 4.8 Hz, 2H), 4.14 (t, *J* = 7.0 Hz, 1H), 3.63 (m, 2H), 3.49 (m, 2H), 3.32 (m, 2H).

### Extraction of Cellulose Nanofibrils (CNF) from Kraft Pulp and Solvent Exchange

CNFs were produced using an earlier described procedure.^35^ Briefly, the dry pulp was cut and finely ground by using a mill. To evaluate the milling degree, the pulp was diluted to a concentration of 0.24 wt % and analyzed using a Schopper-Riegler measuring vessel, resulting in a grinding degree of 92° SR. Subsequently, the suspension was processed through a microfluidizer (Microfluidics M-100EH-30) featuring two fine chambers with orifice sizes of 400 and 200 μm at a pressure of 15,000 psi, followed by two additional finer chambers with orifices of 200 and 100 μm at 25,000 psi. Finally, the cellulose nanofibers (CNF) were concentrated via centrifugation to produce an aqueous CNF gel with a concentration of 2 wt %.

From the resulting aqueous CNF gel (2 wt %), a solvent exchange was made to DMSO following the same procedure as reported.^36^ Following this, the resulting 1.5 wt % CNF gel in DMSO was obtained.

### Preparation of the CNF-Based Macroinitiators by Esterification

CNFs that were solvent-exchanged were subjected to an esterification reaction to produce a CNF-based macroinitiator (CNF-MI). The same procedure is used as reported elsewhere.^36^ Briefly, 20 g of the CNF gel (1.5% w/w in DMSO) was resuspended in DMSO (100 mL). The suspension was heated to 55 °C, followed by the addition of Imidazole (6 g, 44 mmol). In the meantime, 2-bromo-2-methylpropionic acid (8 g, 24 mmol) was dissolved in 60 mL of DMSO and while stirring, CDI (8 g, 24 mmol) was slowly added. The solution was stirred for 1 h at room temperature. Finally, the solution was slowly added to the CNF suspension. The reaction was stirred for 16 h. All steps were performed under a nitrogen atmosphere. The modified CNF was purified by centrifugation (4000 rpm/20 min). The supernatants were discarded and replaced with DMSO. The purification steps were repeated eight times. Thus, an amount of 16 g of CNF-MI at a concentration of 2 wt % has been obtained.

### SET-LRP Grafting of Fmoc-AEEA onto CNF-MI

A copper wire (diameter = 1 mm, length = 6.25 cm) was immersed in concentrated hydrochloric acid for 10 min, then rinsed first with distilled water, acetone, and finally dried with compressed air. Fmoc-AEEA (5 g, 13.11 mmol) was dissolved in 15 mL of DMSO and added to a suspension of CNF-based macroinitiator (4 g, 1 wt %) in DMSO (30 mL) containing the HCl-treated copper wire. The suspension was degassed via nitrogen purging for 10 min and the temperature was raised to 60 °C. The Me_6_TREN ligand was added, and the reaction proceeded under a nitrogen atmosphere for 16 h. The resulting CNF-*g*-Fmoc gel was obtained by centrifugation (6000 rpm, 20 min). The supernatants were decanted, and the rest was dispersed in fresh DMSO. This was repeated five times.

### Gel Formation

To produce a Fmoc-FF gel, a defined amount of Fmoc-FF was dissolved in 1 mL of DMSO. The following calculation based on Dudukovic and Zukoski^37^ gives the amount of distilled water (DI) water that needs to be added to get a gel (eq 1).
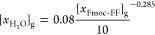
1The volume fraction of DMSO, ϕ(DMSO), has been calculated using the equation shown in eq 2, with *v* for volume in milliliters (eq 2).
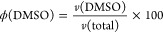
210 μL DI water (10 μL) is added dropwise, and the mixture is mixed for 20 s. After sitting for 10 min, the gelation was tested by flipping the glass vile.

Fmoc-FF gel was made following this procedure: 50 mg of Fmoc-FF dissolved in 1 mL of DMSO. The gelation was started by adding 500 μL of DI water, mixing for 10 s, and letting sit for 10 min after each addition.

“Gel mixing” or “high-intensity mixing” refers to the use of ROTISpeed Agitators, purchased from Carl Roth GmbH + Co. KG, which operates at a speed of between 5000 and 10,000 rpm. The agitator used is a Micropistil-type tool made of stainless steel.

The composite gels were prepared by using the same procedure. An example of a procedure used to make a gel: uCNF/Fmoc-FF_3.89_: 0.09 g of Fmoc-FF was taken and dissolved in 0.5 mL of DMSO, and then 1 g of uCNF (3 wt %) was added to it. This was mixed with a ROTISpeed Agitator for 5 min. Afterward, 1.5 mL of water was added and mixed again. The rest of the gels were made by following the same pattern.

### Gel Printing

A grid model has been designed using Fusion360 and 3D gel printed by pneumatic extrusion using a CELLINK INKREDIBLE 3D bioprinter. One disposable syringe was charged and fitted into the print cartridge. Printability was adjusted by setting the inlet flow rate through the nozzle to achieve a stable and consistent extrusion. The gel was printed with a 0.84 mm diameter conical nozzle with different applied pressures for each gel (18–50 kPa).

### Material Characterization

Attenuated total reflection Fourier transform infrared (ATR-FTIR) spectroscopy was conducted using a Bruker Vector 33 FTIR I18500 PS15 infrared spectrometer. The spectra were recorded with 64 scans across the 4000–500 cm^–1^ spectral region. The spectral resolution is 4 cm^–1^.

Nuclear magnetic resonance (NMR) (Bruker AVANCE III HD 400) operating at 400 MHz was also used to analyze the structures of the compounds. Nuclear magnetic resonance in the solution state with CDCl_3_ as solvent was performed at 25 °C and transferred to NMR tubes with a 5 mm outer diameter.

A calibration standard of varying concentrations of the Fmoc-AEEA monomer was produced on a Lambda 65 UV–vis spectrometer. This spectroscopy is performed in the ultraviolet–visible (UV–vis) range from 380 to 900 nm. The absorption spectra were measured in DMSO as a standard solvent at a resolution of 1 nm in a quartz cuvette of 1 cm. The absorbance of the CNF-*g*-Fmoc sample used for quantitation purposes was recorded by using the same concentration of CNF-MI as a blank.

The rheology tests were carried out with a TA Instrument AR 2000ex and Advantage v5.8.2 software using a 40 mm parallel plate setup and a gap distance of 1000 μm. All of the measurements were done at room temperature, and the gels were deposited on the bottom plate. The sweep frequency range was set between 0.1 and 100 rad s^–1^ and viscoelastic properties were recorded. Yield strength assessment was done by measuring the viscoelastic properties at a 6.28 rad s^–1^ angular frequency to ensure that these were measured within the linear viscoelastic region while increasing the shear rate. A shear stress ramp, ranging from 0.01 to 1100 or 0.01 to 2000 Pa, was applied depending on each material. The rotational-shear viscosity measurements were performed in flow mode, with shear rates varying from 0.01 to 2000 s^–1^. The rotational recovery measurements were conducted to characterize material recovery behavior after 3D printing by applying a low shear rate of 0.01 s for 200 s, then a high shear rate at 895 s for 100 s, and finally a low shear rate of 0.01 s for 200 s.

To determine the dry matter content of the CNF suspension, a certain mass of the suspension was dried at 60 °C overnight and weighed after drying. The following eq 3 was used with *m*_w_ for the weight of the gel in the wet state, and *m*_d_ when dry.
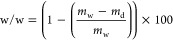
3The morphological properties of modified CNFs and composites were observed via ultrahigh resolution field emission scanning electron microscope (Quanta FEG Type 250, FEI Electron Optics SN:D9122, the Netherlands) at an acceleration voltage of 7 kV and high vacuum conditions using an Everhart–Thornley Detector. The air-dried and supercritical CO_2_ dried samples were mounted onto the stubs using carbon tape. The sample surfaces were then coated with a layer of gold (approximately 5 nm) under an inert atmosphere with a BIORAD SC510 sputtering machine before microscopic imaging.

### Supercritical CO_2_ Drying

The excess DMSO and water were removed from the wet gel after printing by soaking it multiple times in ethanol. Once this was complete, the wet gel was transferred to a high-pressure vessel designed for supercritical CO_2_ drying and extraction. CO_2_ was introduced into the vessel at temperatures and pressures above its critical point (40 °C and 80 bar). The CO_2_ supply was maintained for at least 30 min with a CO_2_ flow of about 3 g min^–1^ to replace and expel most of the solvent. Afterward, the pressure was gradually released, causing the CO_2_ to evaporate and leaving behind a solid aerogel structure. The experimental setup for drying with supercritical CO_2_ is shown in the Supporting Information (Figure S1).

Confocal laser scanning microscopy imaging was performed with a Zeiss LSM900 microscope fitted with an Airyscan2 detector (Zeiss, Oberkochen, Germany), using a Plan-Apochromat 63×/1.4 NA oil immersion objective, a 488 nm solid-state laser diode, an MBS 405/488/561/640 as main beam splitter, 1 AU pinhole aperture, and Nyquist sampling. Fluorescent signals were collected by the Airyscan2 GaAsP detector, while transmitted light was collected by a Multi-Alkali PMT.

The Bruker 500 Avance III HD spectrometer was utilized to acquire ^13^C NMR spectra for all samples. The spectra were recorded at Larmor frequencies of 125 MHz for ^13^C and 500 MHz for ^1^H. To facilitate magic angle spinning (MAS), the samples were placed in 4 mm zirconia rotors and spun at a rotational speed of 8 kHz. For the ^13^C MAS NMR spectra, a ^13^C mutation frequency of 50 kHz and a contact time of 1.5 ms were applied. Fourier transform of the free induction decays (FIDs) was performed to obtain the spectra, and the chemical shifts were referenced to pure tetramethylsilane (TMS).

High-resolution magic angle spinning (HR-MAS) spectroscopy was used to study the molecular composition of CNF-*g*-Fmoc. The experiments were performed by using a Bruker Avance III HD spectrometer equipped with a high-speed magic angle spinning probe. The samples were prepared by placing them uniformly in a 4 mm zirconia rotor. For efficient spinning, the rotor was rotated at a speed of 8 kHz, ensuring efficient homogenization of the sample. For proton (^1^H) spectra, a Larmor frequency of 500 MHz was used, while for carbon-13 (^13^C) spectra, a Larmor frequency of 125 MHz was used. The acquisition parameters for HR-MAS included a 90° pulse length of 4 μs, a relaxation time of 2 s, and a spectral width of 20 ppm. The spectra were processed using appropriate data analysis software, and the chemical shifts were referenced to an internal standard such as tetramethylsilane (TMS).

Small-angle X-ray scattering (SAXS) measurements were conducted at beamline P03 of PETRA III at DESY in Hamburg.^38^ The experiment utilized a beam with a diameter of 25 μm, a wavelength of λ = 0.105 nm, and a sample-to-detector distance (SDD) of 3901 mm. A PILATUS 2 M detector (Dectris, Switzerland) with a pixel size of 172 μm was used, and each pattern was captured with an exposure time of 10 s. All data underwent background correction. The samples were scanned over an area of 2 × 2 mm^2^ with a step size of 0.1 mm; each acquisition takes 1 s to avoid beam damage. Subsequent images were summed and radially integrated for data analysis, allowing for the derivation of intensity *I*(*q*) as a function of wavevector transfer *q*. For data analysis, SASview version 5.0.5 was utilized in conjunction with χ^2^ minimization.^39^ A mask file was applied prior to the integration of the two-dimensional (2D) SAXS pattern. We used a sufficiently long cylindrical form factor to statistically analyze the radius and polydispersity of the Fmoc-FF and the CNF samples detected in the sample. We fitted the distribution of the detected interaction between the Fmoc-FF and CNF-*g*-Fmoc structure domains using a Gaussian distribution.

## Results and Discussion

Due to the ease and efficiency of solvent trigger gelation, it is chosen to produce Fmoc-FF peptide gels. The LMWG is first dissolved in DMSO and then diluted by adding water until gelation occurs. This has been extensively reported previously and has enabled the generation of rigid gels possessing storage moduli as high as 10^4^ Pa, even at concentrations as low as 0.01 wt % of Fmoc-FF.^37,40^ Dudukovic et al.^41,42^ were able to establish a concentration range under which a Fmoc-FF solution in DMSO forms gels upon the addition of water. The resulting curve shows a power law of behavior, allowing the extraction of eq 1 and determining the volume needed to produce the desired gels (eq 1). Based on that, the required amount of water to trigger gelation is determined and gradually added. Fmoc-FF gels obtained are 3.4 wt % and a DMSO volume ratio of 0.66.

Nolan and colleagues^21^ were successful in developing 3D printable Fmoc-FFs gels via solvent switching method. This approach proved to be more effective than the pH change method for printing because of the formation of spherical domains of tightly packed primary fibrils and fibrils. The gel ink used in this study has a lower DMSO volume content (0.45) and is more concentrated (5 mg mL^–1^). Solvent–gel and solvent–solvent H-bonds were found to be the main driving forces behind gelation, and higher water content leads to increased levels of H-bond formation. The print resolution of these inks is quite satisfactory; hence, the purpose of our study is to evaluate if similar or improved 3D-print quality could be achieved by using CNF as an additive.

### Setting the Appropriate Ratio for the Gel Formulation

To set up the proper gel formulation, e.g., the ratio of each component, unmodified CNF (uCNF) is first used due to its accessibility and availability, allowing for easy application. Following the mechanical defibrillation of the cellulose fibrils, the isolated unmodified cellulose nanofibrils undergo solvent exchange from water to DMSO, making them ready for gel preparation. Therefore, two distinct gels are prepared by mixing different ratios of Fmoc-FF and CNF while using identical final concentrations and the same volume ratio of DMSO/H_2_O. The contents of each component are calculated according to the solid weight of each material (for CNF, this is done using eq 3). To prepare the gel, Fmoc-FF is first dissolved in DMSO, then CNF, already suspended in DMSO, is added, the mixture is homogenized, and a certain volume of water is finally added (eqs 1 and 2). The characteristics and properties of the prepared gels, as well as mechanical parameters, are shown in Table 1.

**Table 1:** Summary of the Characteristics of CNF/Fmoc-FF Composite Gels with Different Ratios

[Fmoc-FF/CNF] ratio	concentration (wt %)	ϕ(DMSO)	*G*′ (Pa)	*G*′′ (Pa)	pressure applied during 3D printing (kPa)
75/25	2.67	0.66	14,530	2848	31
50/50	2.67	0.66	7772	1021	15

The mechanistic measurements indicate that the higher peptide content gel has a higher loss and storage modulus if compared to the lower peptide content gel. A square grid model with a size of 1.75 × 1.75 × 2 cm^3^ and with four holes is selected to assess each gel’s printing resolution (Figure S2). Both gels are 3D printed and none of those gels did produce a high-quality print resolution. Even with an increase in the CNF content in the gel, no significant improvement in the print could be noticed. The mechanistic rheological measurements are performed at the same concentrations and DMSO volume ratios. The results indicate that the gel, containing a higher proportion of Fmoc-FF, displays up to twice as high mechanical properties. Therefore, it was agreed that a ratio of 75% Fmoc-FF and 25% CNF would be used in the study based on these considerations and the fact that Fmoc-FF is commercially available and inexpensive, unlike CNF, which must be prepared.

### Straightforward Method for Producing Gel Composite with Unmodified CNF (uCNF)

**Figure 1:**
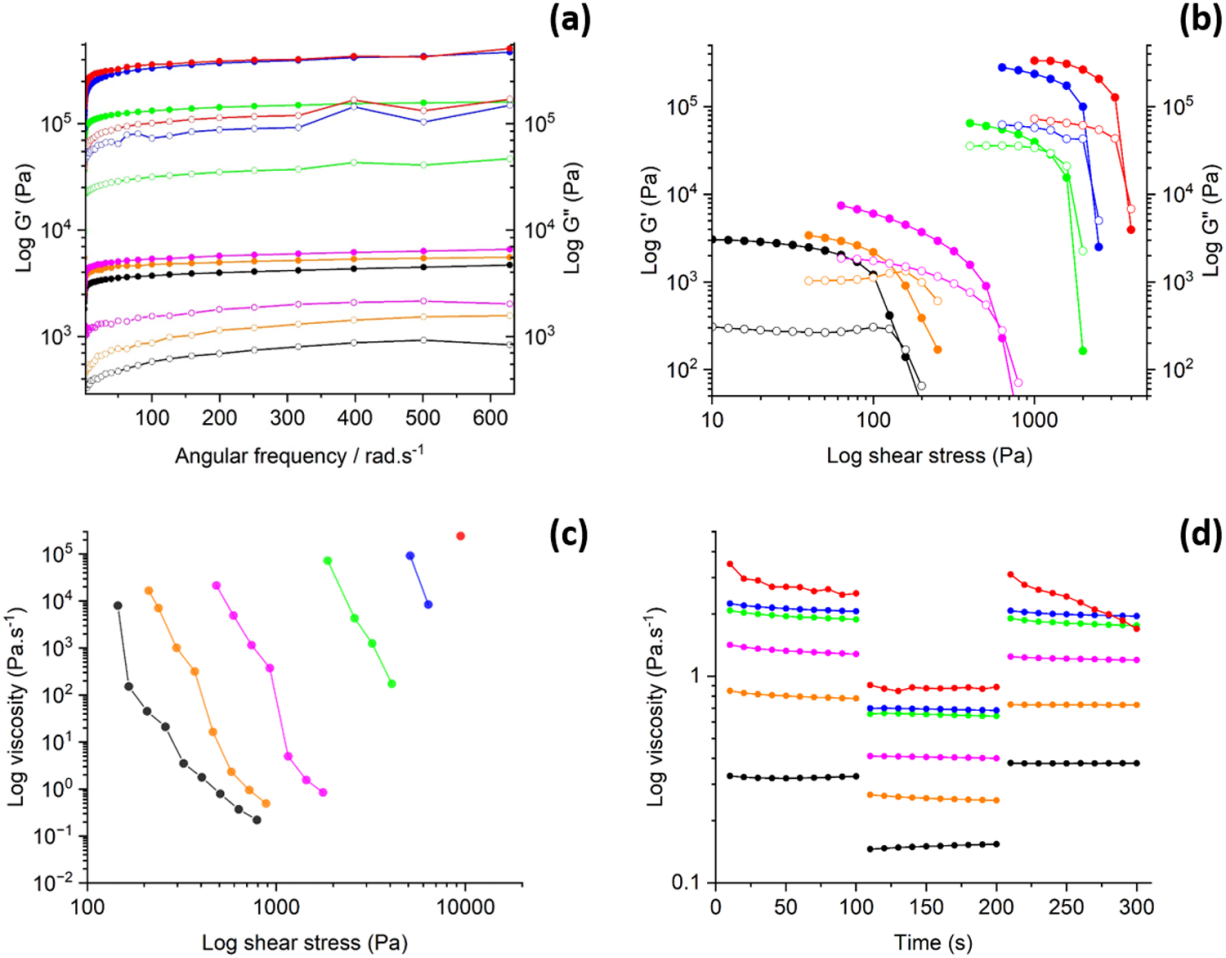
(a) Data of viscoelastic properties of composite gels; (b) dynamic modulus under increasing stress to determine yield stress; (c) shear stress ramp data; (d) recovery tests with small (200 s^–1^) and large (800 s^–1^) pressure applied to gels; colors code: uCNF/Fmoc-FF_7.50_: black, uCNF/Fmoc-FF_6.40_: orange, uCNF/Fmoc-FF_6.20_: magenta, uCNF/Fmoc-FF_5.74_: green, uCNF/Fmoc-FF_5.10_: blue, uCNF/Fmoc-FF_3.89_: red.

As the appropriate ratio of both components is now selected, a series of uCNF/Fmoc-FF gels with various dilutions and concentrations is made using the technique described above. It is well-known that depending on the method of gel preparation, the amounts of solvents, and the conditions, various properties can be obtained.^19^ Their characteristics and properties, as well as mechanical parameters, are shown in Table 2, and the viscoelastic data on a wide range of angular frequencies are shown in Figures 1a,b and S6. The determination of yield stress is made by the measurement of the viscoelastic properties while increasing the shear stress, and the result is shown in Figure 1c. The gels are designated from uCNF/Fmoc-FF_7.50_ to uCNF/Fmoc-FF_3.89_ in the ascending order of their dilution in water. uCNF/Fmoc-FF_7.50_ is the product obtained without water. Its mechanical performance is the weakest with a relatively low storage, loss modulus, and yield stress. This is explained by the absence of nucleation and consequently, gelation of Fmoc-FF, and hence its properties only mirror the ones generated from the uCNF. Upon the addition of water, a mild effect on strength can be observed through a slight increase in their relative viscosity and their viscoelastic properties (storage and loss modulus). A notable increase is observed, with samples uCNF/Fmoc-FF_6.40_ and uCNF/Fmoc-FF_6.20_ indicating the gel point. This point is probably related to the moment when the fragments of Fmoc-FF start to nucleate in large clusters, unable to dissolve again, creating a structure based on the lateral π–π stacking of antiparallel β-sheets of Fmoc-FF, as previously described by Ulijn et al.^43^ Higher dilution (starting from uCNF/Fmoc-FF_5.74_) would bring in more protons allowing the establishment of a larger hydrogen bonding network, and therefore greater hydrophobic attraction, which automatically leads to a constant enhancement of the physical properties (storage, loss modulus, and yield stress). An exponential increase in strength is noted from uCNF/Fmoc-FF_5.74_ onward through to uCNF/Fmoc-FF_3.89_. This indicates that a larger reinforced intramolecular 3D network is being established, reflecting the influence of Fmoc-FF. These data prove that the driving force of the gelation is the addition of water which plays a key role in making the robust 3D network.

**Table 2:** Summary of the Characteristics of uCNF/Fmoc-FF Composite Gels^a^

	concentration (wt %)	ϕ(DMSO)	*G*′ (Pa)	*G*″(Pa)	yield stress (Pa)	pressure applied for printing (kPa)
uCNF/Fmoc-FF_7.50_	7.50	1	3172	385	147	15
uCNF/Fmoc-FF_6.40_	6.40	0.83	4066	547	152	17
uCNF/Fmoc-FF_6.20_	6.20	0.80	4491	1203	587	70
uCNF/Fmoc-FF_5.74_	5.74	0.74	105,400	24,130	1259	15^c^
uCNF/Fmoc-FF_5.10_	5.10	0.66	233,800	55,850	2074	18^c^
uCNF/Fmoc-FF_3.89_	3.89	0.50	274,900	70,000	3268	unprintable^b^
uCNF/Fmoc-FF_3.89_, after mixing	3.89	0.50	39,190	6947	594	27^c^

a ϕ: volume ratio.

b Gel unprintable even after 100 kPa applied pressure.

c After 5 min of mixing.

In addition, some supplementary and complementary rheological measurements are performed. The measurement is now conducted by measuring the viscosity recovery against increasing shear stress (Figure 1d). The compounds displayed a shear-thinning effect, explained by the decrease of the viscosity while raising the shear stress. This is a primordial requirement condition for pneumatic 3D printing. The behavior of the gels before and after printing is assessed, with subjection to a high shear rate over a period and recovery to the low shear rate and examining material behavior. Their behavior is suitable for printing since the recovery to their initial viscosity after removal of the shear rate is noticed. All gels showed some viscosity recovery upon returning to the initial shear rate, signifying their state of equilibrium. As can be seen, uCNF/Fmoc-FF_3.89_ displays somewhat of a drop-in viscosity within the test measures, which is due to it acting closer to a solid rather than a gel and tends to creep out of the measurement area, inducing a misleading drop in viscosity.

### Advanced Method for Producing the Gel Composite: Fmoc-AEEA Modified CNF (CNF-*g*-Fmoc)

**Scheme 2:**
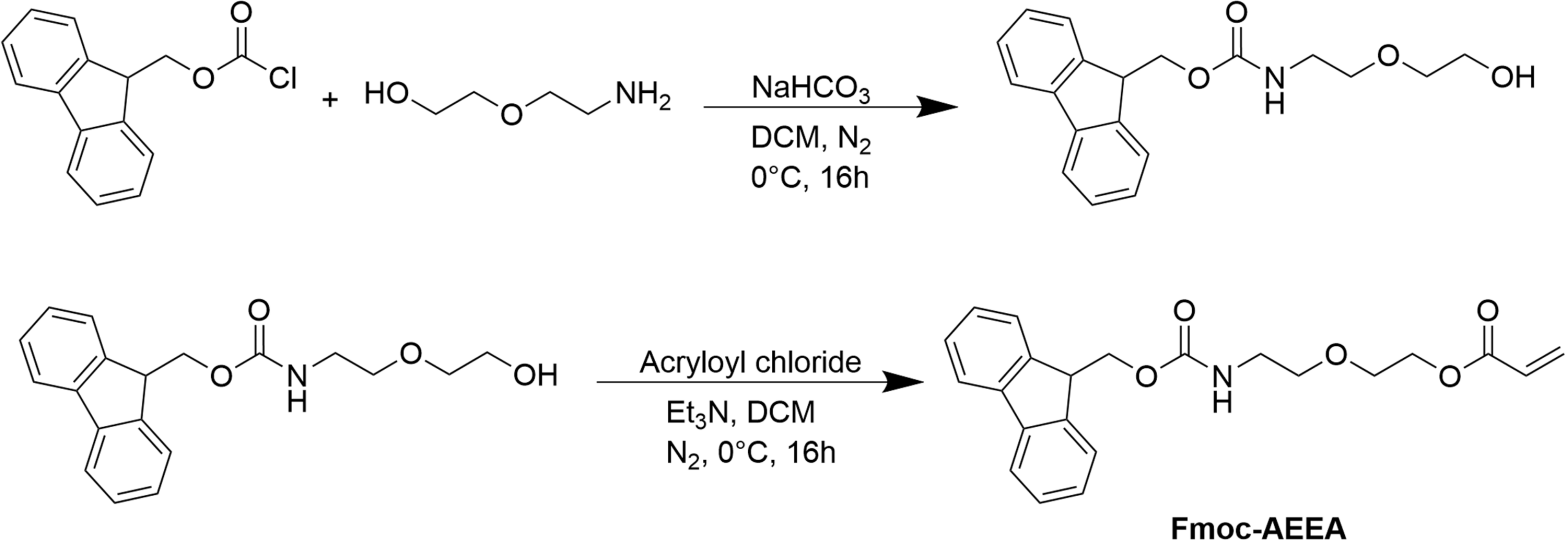
Synthetic Scheme for the Fmoc Monomer (Fmoc-AEEA)

Having established that uCNF does interfere in the self-assembly of the Fmoc-FF moieties, resulting in gels that possess peptide-like properties on their own, it would be appropriate to investigate whether modified cellulose would provide an advantage. The modification of nanocellulose fibrils has been extensively studied and implemented to alter its properties allowing for improved compatibility with matrixes.^44−46^ The envisaged modification consists of introducing fluorenylmethoxycarbonyl (Fmoc) moieties onto the CNF surface. This modification is likely to result in additional hydrogen bonding and π–π supramolecular bonds with the Fmoc-FF matrix. The π–π stacking refers to the interactions between aromatic rings with π systems.^47,48^ These interactions are important for self-assembly, superstructure formation, and system stability. They can take on various forms such as sandwiched and T-shaped conformations. In lignin for example, a predominance of T-shaped stacking interactions has been suggested by SAXS measurements.^49,50^ The CNF modification is done through a SET-LRP type polymerization with a CNF-based macroinitiator and a monomer bearing the Fmoc group. The Surface-Initiated Single-electron transfer living radical polymerization (SI-SET-LRP) is a versatile, extensively documented technique for the controlled synthesis of well-defined polymer layers. It permits precise control over polymer architecture, surface properties, and performance.^51,52^ The monomer was synthesized in a two-step process, and the reaction pathway is shown in Scheme 2. First, 2-(2-aminoethoxy)ethanol is reacted with Fmoc-Cl in basic medium conditions. Next, the as-prepared 2-(*N*-Fmoc-2-aminoethoxy) ethanol is then reacted with acryloyl chloride in the presence of triethylamine in dichloromethane, yielding the 2-(*N*-Fmoc-2-aminoethoxy)ethyl acrylate (Fmoc-AEEA).

**Figure 2:**
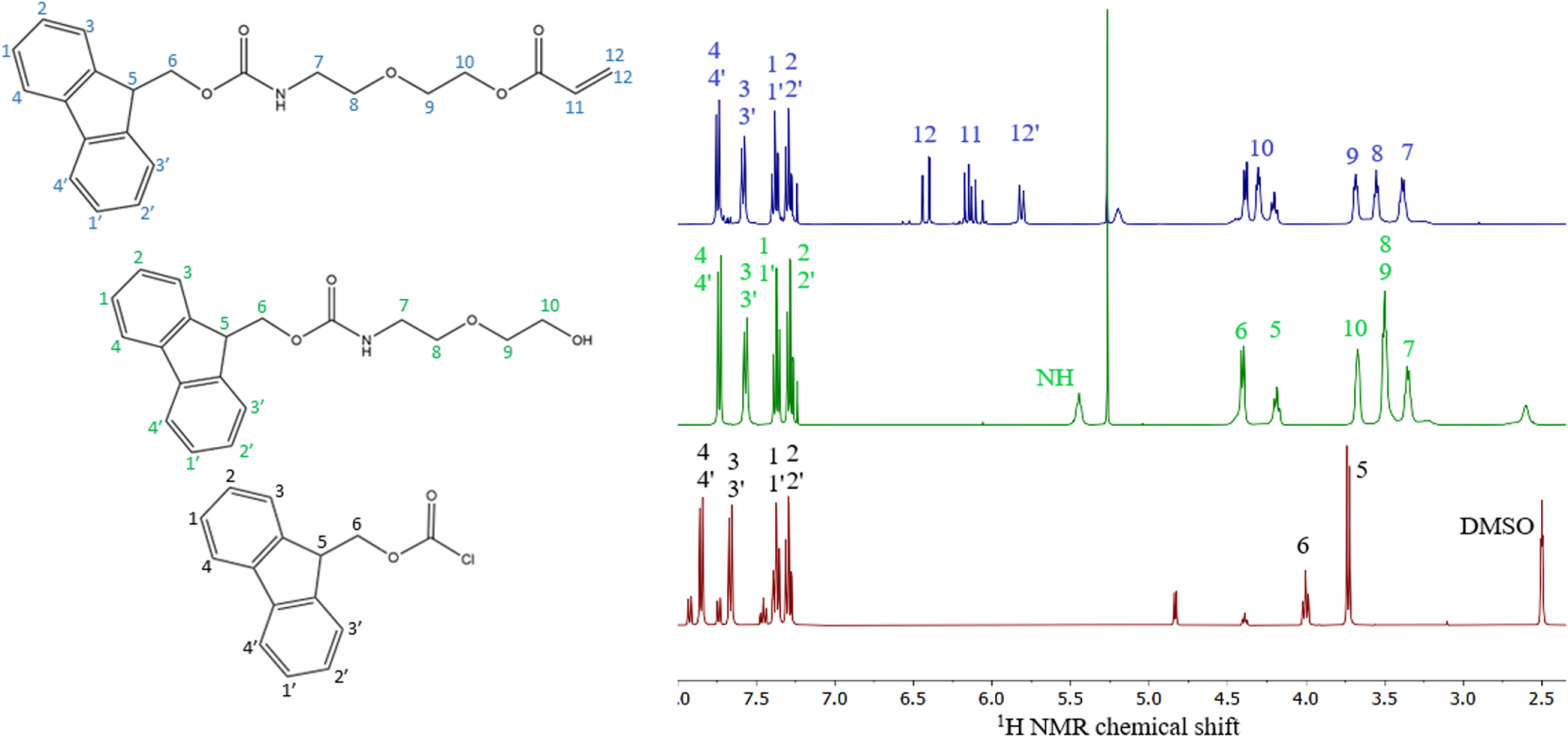
^1^H NMR spectrum of monomer synthesis; red: Fmoc-Cl; green: 2-(*N*-Fmoc-2-aminoethoxy) ethanol; blue: 2-(*N*-Fmoc-2-aminoethoxy)ethyl acrylate.

Nuclear magnetic resonance (NMR) spectra of the starting molecule (Fmoc-Cl), 2-(*N*-Fmoc-2-aminoethoxy) ethanol, and Fmoc-AEEA are shown in Figure 2. The structure of the intermediate can be confirmed by the appearance of NH (5.27 ppm), 7 (3.40 ppm), 8/9 (3.55 ppm), and 10 (3.72 ppm) signals originating from 2-(2-aminoethoxy)ethanol. Ultimately, the monomer structure has been confirmed by the occurrence of the signals 11 (6.08 ppm), 12 (6.34 ppm), and 12′ (5.75 ppm), which correlate with the acrylic function.

**Scheme 3:**
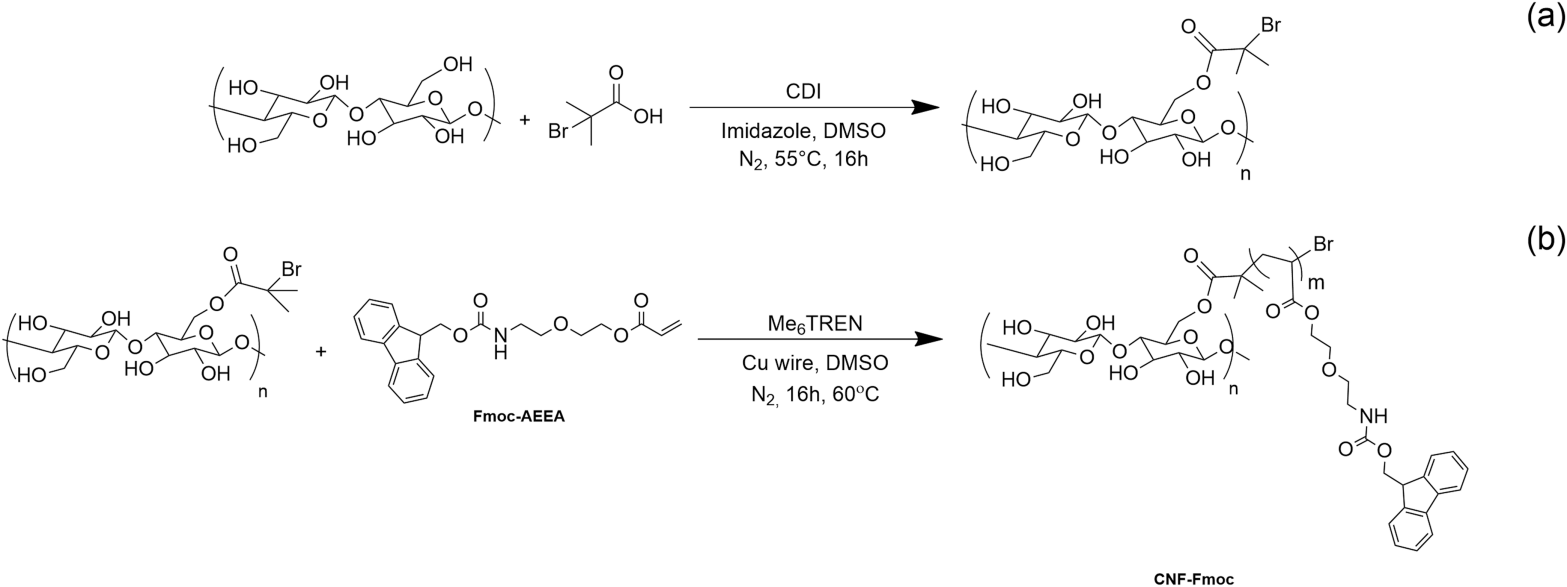
(a) Synthetic Process of the CNF-Based Macroinitiator (CNF-MI), (b) Graft Polymerization of CNF-Based Macroinitiator with Fmoc-AEEA via SET-LRP, Yielding CNF-*g*-Fmoc

Introducing Fmoc functions onto cellulose nanofibrils first requires their chemical modification to convert them into initiators for the polymerization reaction. This is done following a previously described method.^35,36^ The procedure steps can be resumed as follows: a solvent exchange is necessary from water to DMSO after the CNF’s extraction procedure (through mechanical disintegration and microfluidization). Second, an esterification reaction is carried out to convert the CNFs into CNF-based macroinitiators (CNF-MI). A reaction between hydroxyl groups present on the CNF surface is conducted with 2-bromoisobutyric acid in a CDI-promoted basic medium at 55 °C. The grafting of the Fmoc fraction is done through surface-initiated single electron transfer living radical polymerization (SI-SET-LRP).^51,52^ The macroinitiator triggers a controlled chain polymerization reaction of the monomer involving Cu(0) and a tetradentate tertiary amine ligand (Me_6_TREN) in DMSO. Both reactions are depicted in Scheme 3. The modified CNF (CNF-*g*-Fmoc) is finally obtained as a bright yellow gel with a 2–3 wt % concentration by weight obtained by centrifugation.

Fourier transform infrared (FTIR) spectroscopy analysis has been performed to characterize the Fmoc polymer grafting onto the CNF. Cellulose nanofibrils macroinitiator (CNF-MI), Fmoc-AEEA, and CNF-*g*-Fmoc are, respectively, measured, and the spectra are shown in Figure S3. The characteristic signals of CNF-MI exhibit a band located at 3320 cm^–1^ (O–H), 2950 and 2895 cm^–1^ (C–H), 1430 cm^–1^ (C–H), 1161 cm^–1^ (C–O–C), and a carbonyl group of macroinitiator at 1733 cm^–1^ (C=O). For the Fmoc-AEEA monomer, it is possible to identify bands at 1717 cm^–1^ (C=O), 1141 cm^–1^ (N–H), and 738 cm^–1^ (C=C), for instance. For the Fmoc-*g*-CNF, the bands of the monomer merge within the CNF bands, and are weaker and, therefore, are more difficult to distinguish. Some additional analysis must be performed to clarify the matter and confirm the modification of CNF.

**Figure 3:**
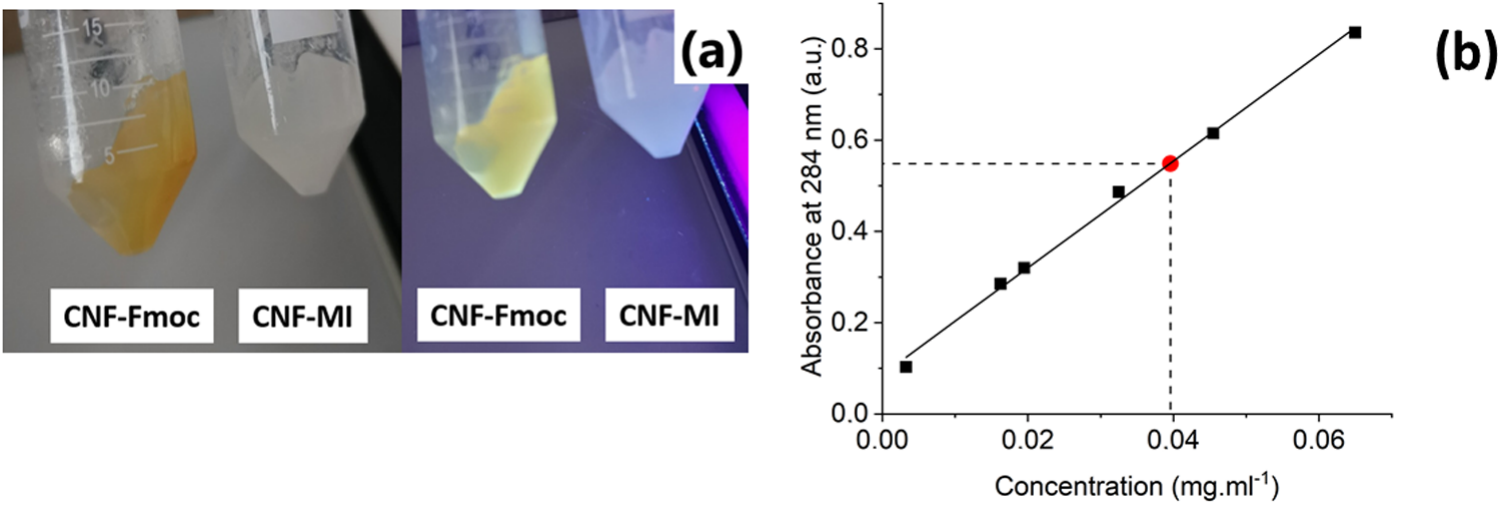
(a) Photographs of CNF-*g*-Fmoc and CNF-MI before UV exposure and after exposure at 366 nm. (b) Calibration curve obtained from Fmoc-AEEA solutions at 284 nm and determination of the unknown concentration for CNF-*g*-Fmoc depicted by the red dot.

Fmoc-Cl is known for having absorption bands located at 210 and 260 nm,^53,54^ while characterizing this molecule through UV–visible spectroscopy. After producing the Fmoc monomer (Fmoc-AEEA), the absorption bands were shifted to the region 280 to 310 nm (see, Figure S4, Supporting Information). In this region, the CNF does not have specific absorption bands. After grafting the Fmoc-AEEA monomer onto the CNF (yielding the CNF-*g*-Fmoc), those new absorption bands appear in the CNF-*g*-Fmoc spectrum in the region from 280 to 310 nm (Figure S4). The modified CNF is seen to emit light in the visible range in comparison to the initial unmodified CNF. These peaks correspond to Fmoc-AEEA, easily observable by the superposition of these spectra, thus supporting the assumption of their presence in the structure. To quantify Fmoc-AEEA content in the CNF-*g*-Fmoc sample, a broad array of solutions containing different concentrations of Fmoc-AEEA have been measured, and the graphical plot of the absorbance at 284 nm, which was selected as it is identical in both samples, against concentration is displayed in Figure 3b. The data reveals a proportionality of absorbance and concentration of Fmoc-AEEA and hence the amount of Fmoc-AEEA incorporated into CNF-*g*-Fmoc can be quantified. A 0.2 mg mL^–1^ concentrated CNF-*g*-Fmoc solution resulted in an absorbance of 0.55, equivalent to a Fmoc-AEEA concentration of 0.04, representing 20% of the total content of the structure.

Since there is evidence of cellulose modification, it is possible to further support this by solid-state CP/MAS ^13^C NMR, and the resultant spectrum is shown in Figure S5a. The characteristic peaks of the chemical groups of cellulose, such as the C1 anomer (100–110 ppm), C4 (80–90 ppm), C6 (60–70 ppm), and C2, C3, C5 (70–80 ppm) can be found on the spectrum.^55^ Peaks relating to the Fmoc-AEEA fragments are expected to be located around 120–145 ppm,^56^ but in this case, no corresponding peaks were detected. It is possible to distinguish a very weak peak around 120–135 ppm, which may correspond to Fmoc-AEEA functions. However, this is insufficient to provide evidence of the modification. Therefore, a high-resolution magic angle spinning (HR-MAS) NMR is performed, and the spectrum is shown in Figure S5b. One of the main advantages of this technique in this context is that it is suitable for swelled samples (in gel form) with high resolution and sensitivity.^57^ CNF-*g*-Fmoc was dried and swollen in DMSO-*d*_6_. The ^1^H NMR peaks are tiny compared to the solvent peaks, which can be attributed to irreversible aggregation of the cellulose nanofibrils during the drying process, and therefore, they do not swell easily and are not easily dispersed, a common characteristic of cellulose materials.^58,59^ While it is difficult to discern the peaks corresponding to CNF, it can be ascertained that there are peaks associated with the Fmoc-AEEA functionality in the 7–8 ppm region.

In the same procedure as previous gels (uCNF/Fmoc-FF), the same concentration and volume ratio of DMSO have been used, while only CNF has been replaced by CNF-*g*-Fmoc, a new series of composites resulted and named CNF-*g*-Fmoc/Fmoc-FF_*x*_, with *x* the concentration of the gel. The mechanistic properties are also measured through rheology, and the result is shown in Table 3.

**Table 3:** Summary of the Characteristics of Composites Prepared Using CNF-*g*-Fmoc^a^

	concentration (wt %)	ϕ(DMSO)	*G*′ (Pa)	*G*″ (Pa)	yield stress (Pa)	pressure applied for printing (kPa)
CNF-*g*-Fmoc/Fmoc-FF_7.50_	7.50	1	14.43	2.569	0.32	unprintable^b^
CNF-*g*-Fmoc/Fmoc-FF_6.40_	6.40	0.83	30.17	5.203	2	unprintable^b^
CNF-*g*-Fmoc/Fmoc-FF_6.20_	6.20	0.80	451.4	56.59	20	12
CNF-*g*-Fmoc/Fmoc-FF_5.74_	5.74	0.74	2537	406.9	146	43
CNF-*g*-Fmoc/Fmoc-FF_5.10_	5.10	0.66	95,300	27,460	1268	25^c^
CNF-*g*-Fmoc/Fmoc-FF_3.89_	3.89	0.50	452,400	80,680	7012	unprintable^c^

a ϕ: volume ratio.

b The gel is too thin.

c After 5 min mixing with a mixer.

**Figure 4:**
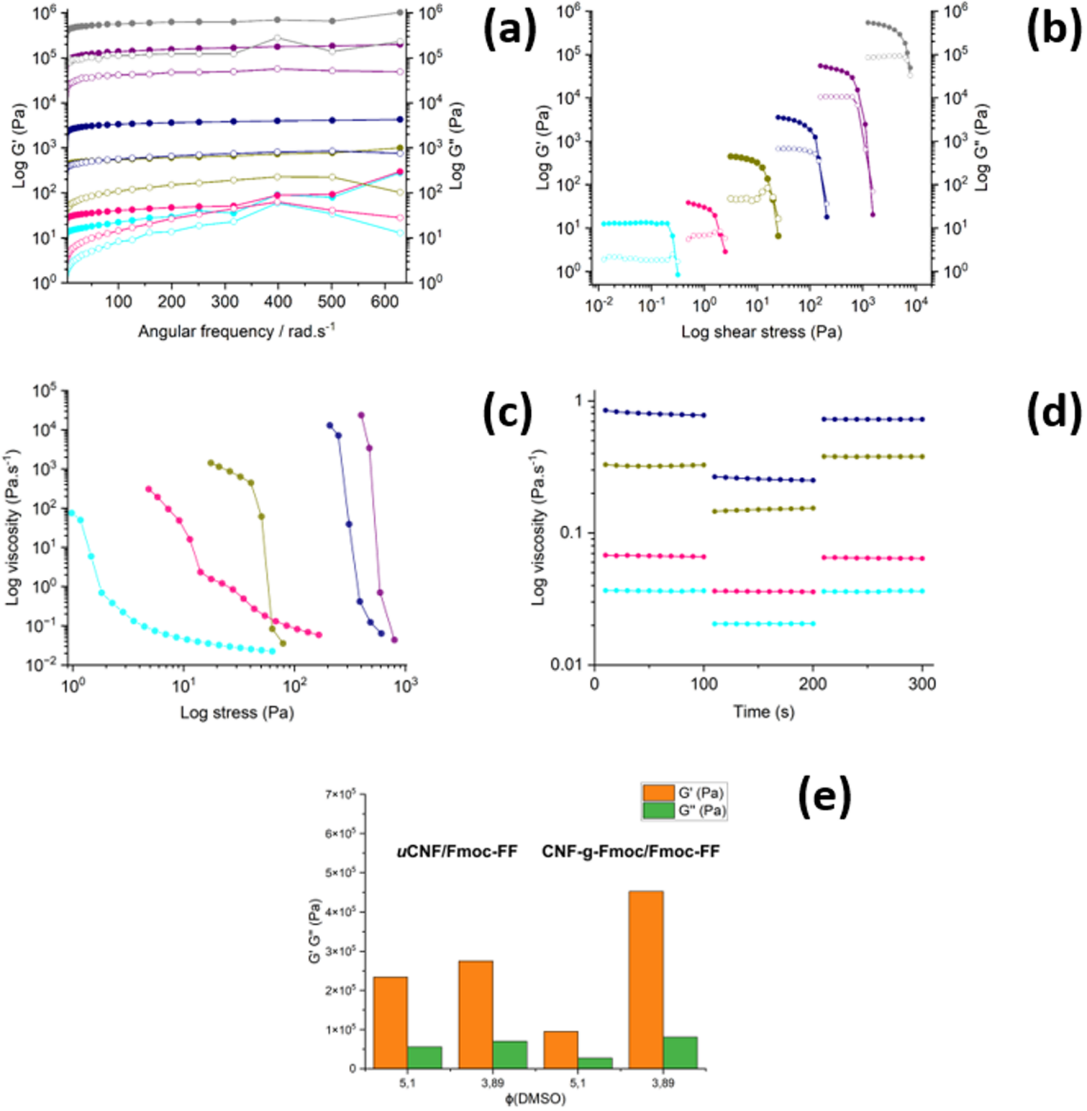
(a) Viscoelastic properties data of composite gels. (b) Dynamic modulus under increasing stress to determine yield stress. (c) Shear stress ramp data, CNF-*g*-Fmoc/Fmoc-FF_3.89_ data are not represented. (d) Recovery tests with small (200 s^–1^) and large (800 s^–1^) pressure applied to gels, at different times simulating extrusion conditions that the materials are subjected to. (e) Bar charts showing the loss and storage modulus results of the different gels. Colors code: CNF-*g*-Fmoc/Fmoc-FF_7.50_: cyan, CNF-*g*-Fmoc/Fmoc-FF_6.40_: pink, CNF-*g*-Fmoc/Fmoc-FF_6.20_: dark yellow, CNF-*g*-Fmoc/Fmoc-FF_5.74_: navy, CNF-*g*-Fmoc/Fmoc-FF_5.10_: purple, CNF-*g*-Fmoc/Fmoc-FF_3.89_: gray.

As a preliminary observation, all the gels (except uCNF/Fmoc-FF_3.89_) produce using CNF-*g*-Fmoc have significantly weaker mechanical characteristics than gels produced using uCNF, which is quite unexpected at first glance. There is a sharp contrast between CNF-*g*-Fmoc/Fmoc-FF_5.74_ and CNF-*g*-Fmoc/Fmoc-FF_5.10_, where a rapid increase in the mechanical properties is observed, thus, pointing out that more water is needed to build up a more stable structure. This could well be due to the increased aggregation effect with CNF-*g*-Fmoc due to the additional interactions (hydrogen bond and π–π interactions). Nevertheless, CNF-*g*-Fmoc/Fmoc-FF_3.89_ has far superior properties (storage modulus and yield strength) to that of its counterpart, uCNF/Fmoc-FF_3.89_, with the same concentration and volume DMSO ratio. This indicates the extra interactions taking place resulting in an overall stronger gel that is closer to being a “solid” than to a “gel”. Figure 4 presents a graphical representation of the detailed rheological results obtained from the same methods used previously for the uCNF/Fmoc-FF composite gels. For the measurement of rotational recovery, CNF-*g*-Fmoc/Fmoc-FF_3.89_ could not be measured, as there was a gradual decrease in viscosity under the steady shear rate, rendering them unusable. For the sake of comparison and visualization of the mechanistic outcomes (Figure 4e), a bar chart is employed to depict the loss and storage modulus results of the gels at their highest dilutions.

### Confocal Laser Scanning Microscopy

**Figure 5:**
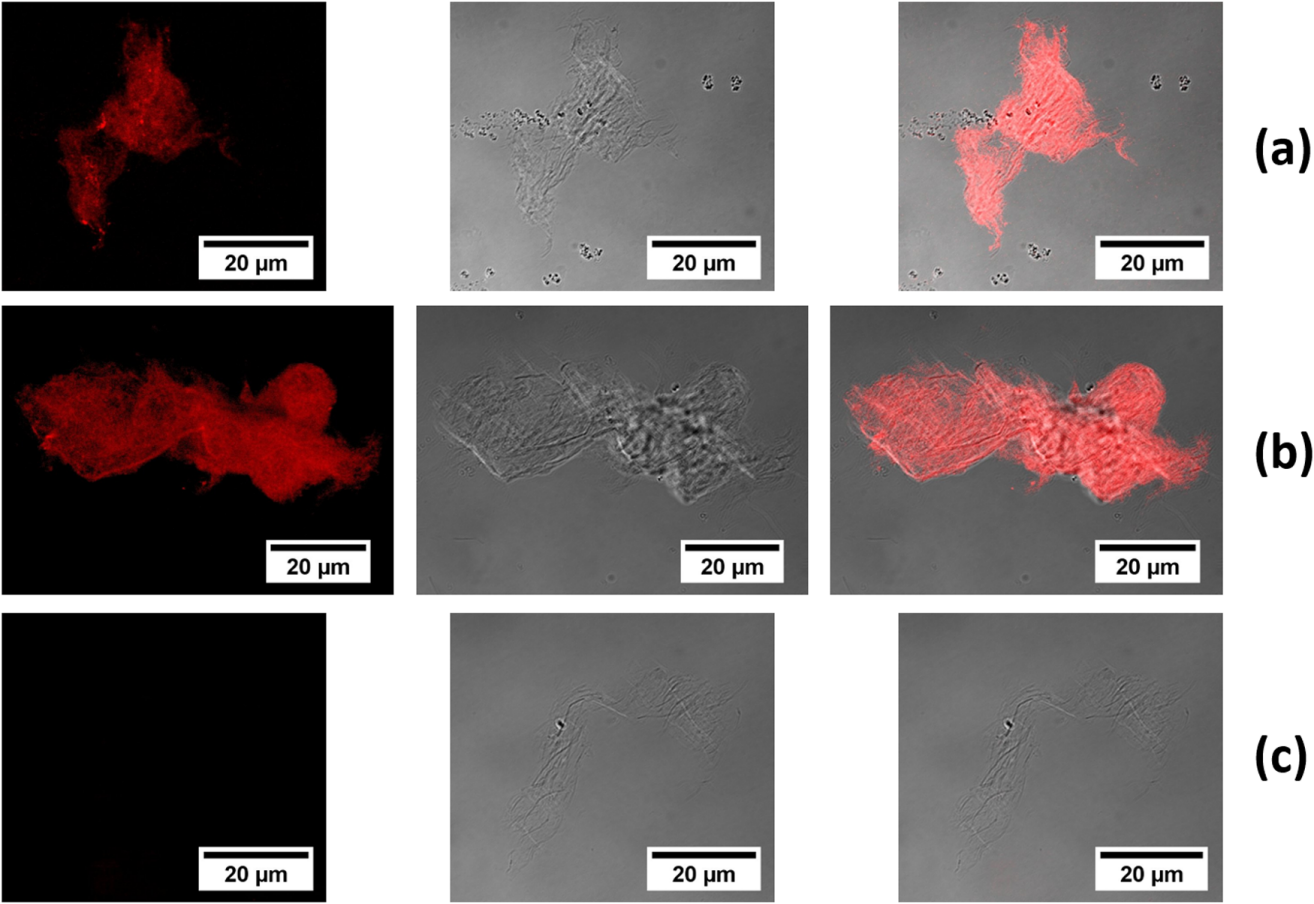
CLSM images (fluorescence, bright-field, combined overlay) of (a) CNF-*g*-Fmoc, (b) CNF-*g*-Fmoc/Fmoc-FF, and (c) uCNF/Fmoc-FF. Excitation wavelength: 488 nm.

As it was observed that cellulose modified with Fmoc groups (CNF-*g*-Fmoc) is fluorescent when excited with UV light, the sample was characterized by using confocal laser scanning microscopy (CLSM). Dried samples of CNF-*g*-Fmoc, CNF-*g*-Fmoc/Fmoc-FF composites are now characterized using CLSM, and the results are presented in Figure 5. The fluorescence images are obtained with a 488 nm excitation wavelength. The resulting images, fluorescence (Figure 5, left), bright field (Figure 5, middle), and the combined overlay fluorescence-bright-field images (Figure 5, right) are acquired using a Plan-Apochromat 63×/1.4 NA oil immersion objective. The fluorescence images revealed the presence of a fluorescence signal for the samples CNF-*g*-Fmoc and CNF-*g*-Fmoc/Fmoc-FF, while, as expected, no fluorescence signal was observed for the sample uCNF/Fmoc-FF. By looking at the combined overlay fluorescence-bright-field images, it can be seen that all of the luminescent spots can only be observed where the fibrils are. The perfect combination of the overlay bright field/fluorescence proved that the fluorescence spots come from the modified surface of the fibrils, and the signal is homogeneously distributed over the CNF surface.

### Morphological Analysis

**Figure 6:**
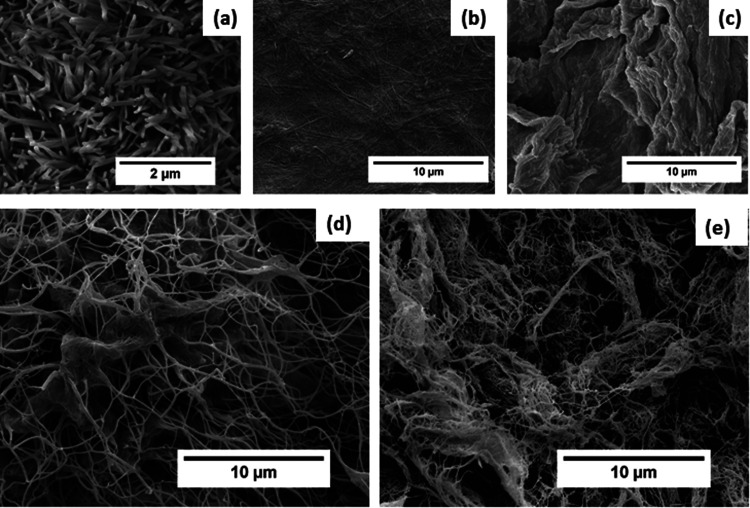
Field emission scanning electron microscopic (FESEM) images. (a) Fmoc-FF sample, air-dried. (b) **u**CNF sample, air-dried. (c) CNF-*g*-Fmoc, air-dried (d) **u**CNF/Fmoc-FF_5.10_, supercritical CO_2_ dried. (e) CNF-*g*-Fmoc/Fmoc-FF_5.10_ supercritical CO_2_ dried.

To achieve a deeper insight into the fibril structure of each sample and to assess the impact of chemical modification on their morphology, samples including Fmoc-FF, CNF (modified and unmodified), and composites were analyzed using field emission scanning electron microscopy (FESEM). Using advanced imaging techniques, high-magnification and resolution imaging was achieved, giving in-depth information about the structure and organization of the fibrillar networks and thereby evaluating the effects of chemical modifications. After air drying, a sample of Fmoc-FF with a ϕ(DMSO) of 0.66 and *c* of 3.89 wt % was examined, and the findings are presented in Figure 6a. The imaging revealed a densely packed network of small, rod-shaped, and twisted nanowire structures with an average fibril diameter of approximately 150 nm. This pattern and fibril diameter are consistent with previous reports in the literature.^60,61^Figure 6b depicts a sample of CNF, which displays an extremely compacted network of fibrils possessing a greater length than the Fmoc-FF. However, the chemical modification, hereby grafting of Fmoc-AEEA onto the cellulose, has led to a noticeable alteration in the appearance of the fibrils (Figure 6c). A thick coating of material is now visible surrounding the fibrils, although some remain discernible in the background. This change in morphology suggests a higher degree of aggregation when compared to unmodified cellulose caused by the introduction of the Fmoc groups, leading to an increase in intramolecular interactions. Figure 6d displays an image of uCNF/Fmoc-FF_5.10_ composite gel (ϕ(DMSO) of 0.66 and *c* of 5.10 wt %) that was subjected to supercritical CO_2_ drying. The drying process resulted in two distinguishable network types: one comprising large, easily identifiable cellulose nanofibrils and the other occupying the spaces not taken up by the former. Based on their dimensions and proportions, it can be deduced that the latter corresponds to the Fmoc-FF nanowires. Interestingly, in contrast to uCNF composites with comparable ratios and dilutions, the drying of the gels made from CNF-*g*-Fmoc proved to be very different. When looking at the SEM pictures of the CNF-*g*-Fmoc/Fmoc-FF_5.10_ (Figure 6e), the structures of both networks are distinguishable, but the size and morphology of the Fmoc-FF nanowires were affected and became smaller, probably as the result of the direct interaction of the modified CNF and the Fmoc-FF. Incorporating small-angle X-ray scattering (SAXS) would reveal the nature of the dominant interactions occurring in the gel and their spacing and provide insight into the nanoscale conformation of the structure.

### Small-Angle X-ray Scattering (SAXS) Measurements of CNF and Modified CNF Species in the Fmoc-FF Matrix

In order to deepen the understanding of the structure of the unmodified CNF and the CNF-*g*-Fmoc encapsulated in the Fmoc-FF matrix, a series of SAXS measurements are performed. SAXS experiments were carried out to characterize the pristine CNF (uCNF) in the Fmoc-FF matrix (annotated as uCNF/Fmoc-FF) and CNF-*g*-Fmoc in the Fmoc-FF matrix (annotated as CNF-*g*-Fmoc/Fmoc-FF). All data were background corrected as described previously.

**Figure 7:**
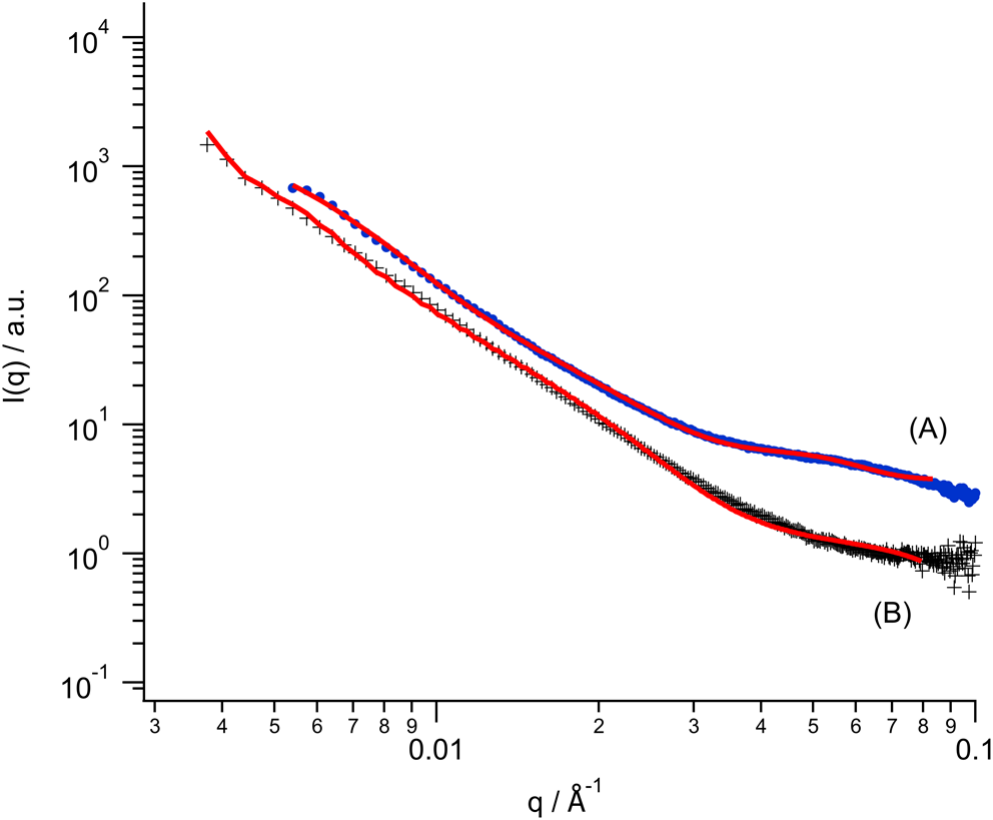
1D-SAXS curves and corresponding fits (red line): (A) CNF-*g*-Fmoc/Fmoc-FF and (B) uCNF/Fmoc-FF.

One-dimensional (1D) SAXS curves *I*(*q*) were calculated by integrating the full 2D SAXS pattern over the full azimuth, and the results are exposed in Figure 7.

The pristine CNF shows a structure with a radius (*R*) of 81 ± 9 nm, which is interpreted as bundles or aggregates of CNFs. Those nanostructures showed a length for the nanofibrils of 752 ± 186 nm. The second contribution comes from the presence of the Fmoc-FF nanowires, showing a structure with a radius of 4 ± 1 nm within a length of 993 ± 424 nm. The absence of a Gaussian band confirmed that the Fmoc-FF and the uCNF do not directly interact physically together and form a network. However, the presence of the uCNF drastically lowers the stiffness of the Fmoc-FF gel.

When looking now at the acquired data for the CNF-*g*-Fmoc/Fmoc-FF, a completely new profile is revealed. The grafting of the Fmoc unit onto the CNF (CNF-*g*-Fmoc) shows a clear change from the initial structure with a new *R* = 15.6 ± 2 nm and a length of 381 ± 62 nm. This structure is attributed to single cellulose nanofibrils. This proves that the presence of the grafted polymer onto the CNF avoids any potential agglomeration of the CNF, e.g., the presence of only isolated CNF, which has already been seen as it is making them extremely stable in suspension. Interestingly, the dimensional structure of the Fmoc-FF nanowire is also influenced by the presence of the grafted polymer onto the CNF due to their respective interaction, with a new *R* = 9.3 ± 1 nm and a length of 495 ± 124 nm. In addition, a new broad structure appears through the presence of a Gaussian band (*q* = 0.0343 Å^–1^), which corresponds to a space length scale of 18.3 nm. This length scale is interpreted as a space where the Fmoc unit of the matrix (Fmoc-FF) directly interacts with the Fmoc unit of the grafted polymer (CNF-*g*-Fmoc) while creating a 3D cross-linked network between the two elements.

### 3D Printing Test

**Figure 8:**
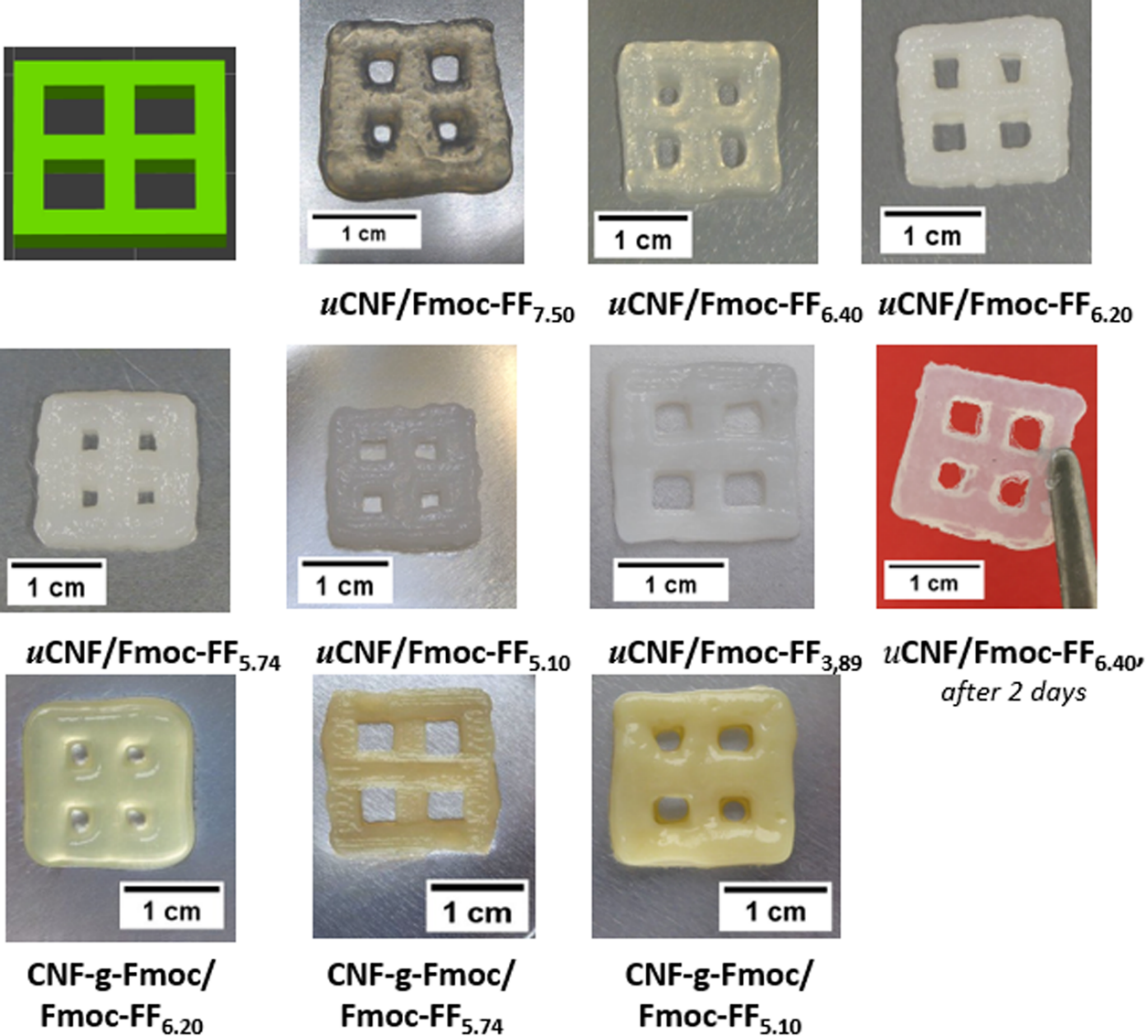
Photographs of the printed models out of *u*CNF/Fmoc-FF and CNF-*g*-Fmoc/Fmoc-FF composite gels.

All different gels are used for printing the grids (see Figure 8). It should be noted that before the water addition, the gels were more translucent and then turned hazy following this addition. This increase in absorbance is representative of the nucleation phase occurring during peptide self-assembly.^17^ No decrease in cloudiness is noted over time, suggesting that the stable formed structures are of sufficient nanosize to scatter light.^62,63^

The uCNF/Fmoc-FF gels are first used for printing, and all gels except for uCNF/Fmoc-FF_3.89_ could be 3D printed. The less diluted uCNF/Fmoc-FF_7.50_ presents a rather poor print quality, and the structure fails to hold. The hypothesis that uCNF/Fmoc-FF_6.40_ had not reached the gelation point claimed above can be confirmed since it remains translucent, e.g., no formation of Fmoc-FF nanostructure. Beyond this point, the compounds became increasingly opaque, suggesting the formation of structures large enough for light to be scattered. An increasing improvement in grid printing quality is observed as the water content of the gel increases. uCNF/Fmoc-FF_3.89_ composite gel, with the highest mechanical properties, exhibited more solid-like behavior if compared to the other gels, which led to difficulties in deforming under the gel 3D-printer extrusion pressure, yielding poor shape homogeneity. Research has shown that greater mixing changes the properties of the gel, improving the homogeneity of the hydrogel and the quality of the 3D-printed structures.^64,65^ The viscoelasticity and yield strength are measured both before and after 5 min of intense mixing, and the results are shown in Figure S3. The mixing for 5 min with an agitator ROTISpeed before printing significantly improved the performance and the print quality. Research has shown that applying high shear homogenization (4000–16,000 rpm) to an acid-gelled soy protein isolate (SPI) significantly reduced particle size and lowered the polydispersity index.^66^ A decrease in the mechanical properties could be noticed, most likely due to the reduction in particle size, as outlined earlier, enabling printing to be carried out. However, due to its concentration, a degree of syneresis under extrusion pressure is observed, rendering the printing challenging. Consequently, uCNF/Fmoc-FF_5.10_ produced the most consistent and best print quality, making it an ideal candidate for further use. The 5 min mixing is also adopted in uCNF/Fmoc-FF_5.10_ to achieve better homogeneity along the entire printing process. Also shown is a print made with uCNF/Fmoc-FF_6.40_, which is left for 2 days under ambient conditions and proved to be solid enough to be handled despite being in gel form. This demonstrates the positive aging effect on the characteristics of the gel.

3D printing is then carried out using the CNF-*g*-Fmoc/Fmoc-FF composites, and the printing performances are also presented in Figure 8. CNF-*g*-Fmoc/Fmoc-FF_7.50_ and CNF-*g*-Fmoc/Fmoc-FF_6.40_ were determined to be unsuitable for 3D printing because their viscosities were too low to provide structural integrity. If now compared to their analogous gels (uCNF/Fmoc-FF_7.50_ and uCNF/Fmoc-FF_6.40_, which are 3D printable), the reduced viscosity and weaker structural integrity likely result from some interactions that are too weak between the CNF-*g*-Fmoc and the Fmoc-FF. CNF-*g*-Fmoc/Fmoc-FF_6.20_ is printable, but the quality of the print is rather unsatisfying, as the outlines are not well-defined. CNF-*g*-Fmoc/Fmoc-FF_5.74_ presents satisfying results as a printable ink. However, by increasing the water content (CNF-*g*-Fmoc/Fmoc-FF_5.10_), a significantly higher clumping was observed, as well as the formation of larger and more robust gel particles, preventing CNF-*g*-Fmoc/Fmoc-FF_5.10_ from printing. In order to be 3d printed, the sample CNF-*g*-Fmoc/Fmoc-FF_5.10_ requires further mixing (5 min) before being printed. Despite being printable after this mixing process, a reduced print quality is observed (Figure 8). CNF-*g*-Fmoc/Fmoc-FF_3.89_ failed to print due to similar syneresis effects, even more marked in this case, as for uCNF/Fmoc-FF_3.89_. In conclusion, CNF-*g*-Fmoc/Fmoc-FF_5.10_ has resulted in more consistent and accurate printing and, hence, is chosen as a suitable candidate for later use.

### Supercritical Drying

**Figure 9:**
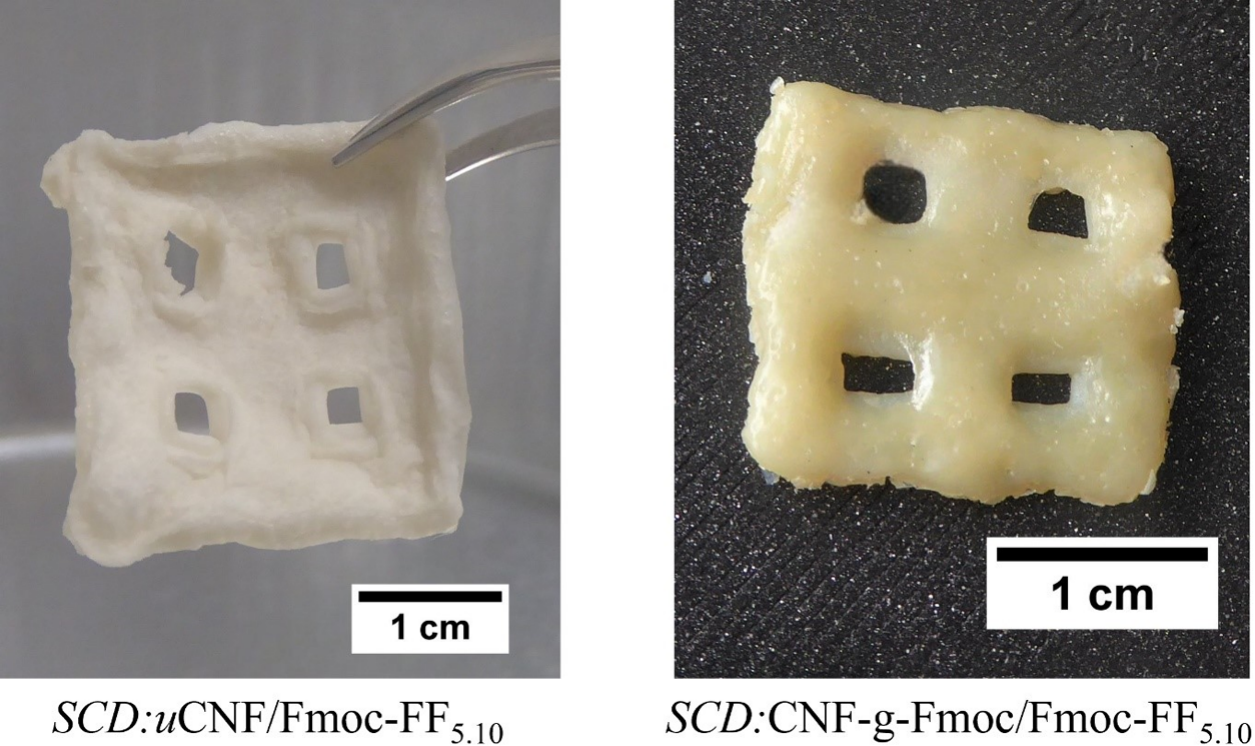
Photographs of the resulting supercritical CO_2_-dried composite gels.

Interest in nanocellulose-based aerogels has grown rapidly in recent years. Their ultralow density, tunable porous structure, and remarkable mechanical properties make them highly attractive for various applications.^67,68^ Supercritical CO_2_ drying is chosen for its ability to effectively preserve the structure and achieve an increased specific surface area. The method allows the structure to be studied more effectively, providing further insight into the eventual nanostructure.^69,70^ Drying is carried out on uCNF/Fmoc-FF_5.10_ and CNF-*g*-Fmoc/Fmoc-FF_5.10_ gels, which are first subjected to a stepwise solvent exchange to ethanol and then to CO_2_. The system is brought to 40 °C and 80 bar pressure to achieve supercritical conditions, and the entire operation is completed in 30 min with a CO_2_ flow of about 3 g min^–1^. Photographs of the aerogels (SCD:uCNF/Fmoc-FF_5.10_ and SCD:CNF-*g*-Fmoc/Fmoc-FF_5.10_) are displayed in Figure 9. It can be noticed that structural integrity has been maintained and very little deformation has occurred.

### Complex Structures Printing

**Figure 10:**
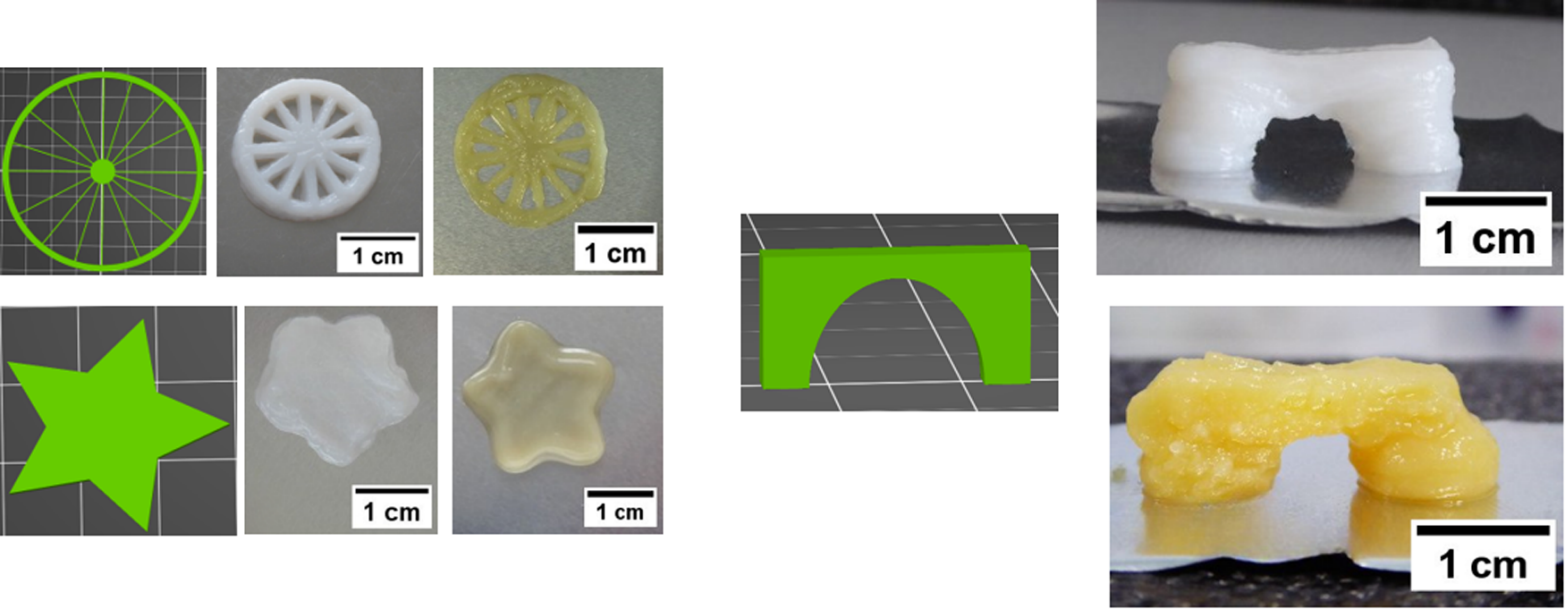
Screenshot from the slicer displaying the 3D model (wheel, star, circle and bridge); photographs of the resulting prints; white gel: uCNF/Fmoc-FF_5.10_ and yellow gel: CNF-*g*-Fmoc/Fmoc-FF_5.10_.

After the printability of those inks was evaluated, the next step was to produce some 3D-printed models with varying degrees of complexity. The quality of these models is assessed based on their fidelity to the original patterns, uniformity of extruded strains, and visual appearance. The selected designs for this study include a 12-spoke wheel, a star, and a bridge, which are shown in Figure 10, along with a screenshot of the model captured from the Slicer and a photo of the print results. For the uCNF/Fmoc-FF composites, uCNF/Fmoc-FF_5.10_ (white) is used, while for the CNF-*g*-Fmoc/Fmoc-FF composite, CNF-*g*-Fmoc/Fmoc-FF_5.10_ (yellowish) is chosen. To ensure the uniformity and consistency of the preparation procedure, both products underwent a rigorous 5-min high-intensity mixing cycle before printing. This allows for a higher degree of monitoring of the result of each print, as any anomalies or fluctuations in the compounding could compromise both the quality and the overall integrity of the finished product.

Both gels are extruded through a cone-tipped pneumatic cartridge with the tip measuring 0.84 mm in diameter, as a smaller cone caused clogging. The fidelity of the circle, star, and ring planar designs is satisfactory with both gels, resulting in a close fit to the model. While there is a modest advantage in print quality with uCNF/Fmoc-FF_5.10_ gel compared to CNF-*g*-Fmoc/Fmoc-FF_5.10_, a lack of uniformity of strains is observed, indicated by the wheel pattern, which may be attributed to the greater mechanical properties of uCNF/Fmoc-FF_5.10_. To demonstrate the superior properties and strength of the gels, a bridge with multiple weak spots was printed with the central deck supported only by two pillars. Fine bridges were produced using both inks, and they were both capable of supporting the load of the deck gels. It can be noticed that the uCNF/Fmoc-FF_5.10_ ink is yielding better results. The results presented here for the CNF-*g*-Fmoc/Fmoc-FF_5.10_ gel were obtained under the best mixing and homogenization conditions. Several alternative techniques were attempted, such as slower mixing, gelation drop by drop, and heating cycles at 40 °C, but no significant improvement was achieved. As a final demonstration of the printing performance, a video of bridge printing was recorded, available in Supporting Information, which shows each filament being deposited and the formation of a 4 mm diameter bridge over two supporting stacks. This new composite gel allows excellent print quality while requiring only a single mixing step and no postprocessing, with models remaining intact throughout the process. This presents a high potential for further exploration to develop novel gels with innovative features.

## Conclusions

In summary, through an extensive examination of Fmoc-FF composites combined with modified and unmodified cellulose nanofibrils (uCNF), a series of high-quality gels with exceptional mechanical properties were developed. Initially, two different ratios of CNF were thoroughly explored, in which the 75% Fmoc-FF ratio proved to be the most appropriate and subsequently chosen. The mechanical properties were evaluated through rheology, and the printability of the initial set of five composites, using uCNF at varying water dilutions, was assessed. Among them, gel uCNF/Fmoc-FF_5.10_ exhibited remarkable print quality and mechanical properties suitable for printing. Subsequently, a similar series of gels was prepared using Fmoc-modified cellulose (CNF-*g*-Fmoc). Two methods were used to modify cellulose, one involving the introduction of a macroinitiator, and the other utilizing SET-LRP to introduce a presynthesized Fmoc moiety monomer in two steps. This was confirmed through solid-state NMR, HR-MAS, and the fluorescence enhancement of CNF-*g*-Fmoc, allowing its visualization under fluorescence microscopy. However, the gel properties in the presence of water differed, exhibiting weaker mechanical properties due to increased gelation, but at significantly higher dilutions. The optimal gel for printing in this scenario was CNF-*g*-Fmoc/Fmoc-FF_5.10_, at the same dilution as that of uCNF/Fmoc-FF_5.10_. The cellulose and composite structures are further examined using FESEM. SAXS experiments characterized both pristine CNF and CNF-*g*-Fmoc in the Fmoc-FF matrix. The results showed that while unmodified CNF does not interact with the Fmoc-FF nanowires, grafted CNF-*g*-Fmoc forms a cross-linked 3D network with Fmoc-FF, significantly altering both structures. Finally, various intricate models, both flat and upright, are successfully printed, showcasing the excellent printing quality of these two gels. These findings underscore the potential of these gels for 3D printing and future applications.

## Supporting Information Available

Video of the 3D-printing process (MP4)
Schematic representation of the experimental setup (P&ID) for supercritical CO_2_ drying (Figure S1); left panel: 3D grid model with 17.5 × 17.5 × 20 mm^3^ dimension; right panel: images of printed grids with the associated ratio below (Figure S2); ATR-FTIR spectrum of pristine Fmoc-AEEA, CNF-MI, and CNF-*g*-Fmoc (Figure S3); UV–vis spectra of Fmoc-AEEA and CNF-*g*-Fmoc (Figure S4); solid-state CP-MAS ^13^C NMR and ^1^H HR-MAS NMR spectrum of CNF-*g*-Fmoc (Figure S5); viscoelastic properties data and dynamic modulus under increasing stress to determine yield stress of *u*CNF/Fmoc-FF_3.89_ before and after mixing (Figure S6) (PDF)
