# Supplementary material for: From Unprintable Peptidic Gel to Unstoppable: Transforming Diphenylalanine Peptide (Fmoc-FF) Nanowires and Cellulose Nanofibrils into a High-Performance Biobased Gel for 3D Printing
Source: ACS Appl Bio Mater. 2025 Mar 7;8(3):2323–39. doi: 10.1021/acsabm.4c01803 (PMC11921036; doi:10.1021/acsabm.4c01803)
Supplement: Supplementary file 2 — mt4c01803_si_002.pdf [file mt4c01803_si_002.pdf]

Supplementary information

From Unprintable Peptidic Gel to Unstoppable: Transforming Diphenylalanine Peptide (Fmoc-FF) Nanowires and Cellulose nanofibrils into a High-Performance Bio-Based Gel for 3D Printing.

Feras Dalloul[a], J. Benedikt Mietner[a], Dhanya Raveendran[a], Shouzheng Chen[c], Enguerrand Barba[a], Dennis M. J. Möck[b], Fabio Hubel[a], Benedikt Sochor[c,d], Sarathlal Koyiloth Vayalil[c,e], Linnea Hesse[a], Andrea Olbrich[b], Jörn Appelt[b], Peter Müller-Buschbaum[f], Stephan V. Roth[c,g] and Julien R. G. Navarro[a,]*

[a]Institute of Wood Science, Universität Hamburg, Haidkrugsweg 1, 22885 Barsbüttel, Germany

[b]Johann Heinrich von Thünen Institute, Federal Research Institute for Rural Areas, Forestry and Fisheries, Institute of Wood Research, Haidkrugsweg 1, 22885 Barsbüttel, Germany

[c]Deutsches Elektronen-Synchrotron DESY, Notkestrasse 85, 22607 Hamburg, Germany

[d]Advanced Light Source, Lawrence Berkeley National Laboratory, 6 Cyclotron Rd, Berkeley, CA 94720, USA

[e]Applied Science Cluster, UPES, Dehradun, Uttarakhand, 248007, India

[f]Technical University of Munich, TUM School of Natural Sciences, Department of Physics, Chair for Functional Materials, James-Franck-Strasse 1, 85748 Garching, Germany

[g]KTH Royal Institute of Technology, Department of Fibre and Polymer Technology, Teknikringen 56-58, 10044 Stockholm, Sweden

Corresponding Author: Email: julien.navarro@uni-hamburg.de

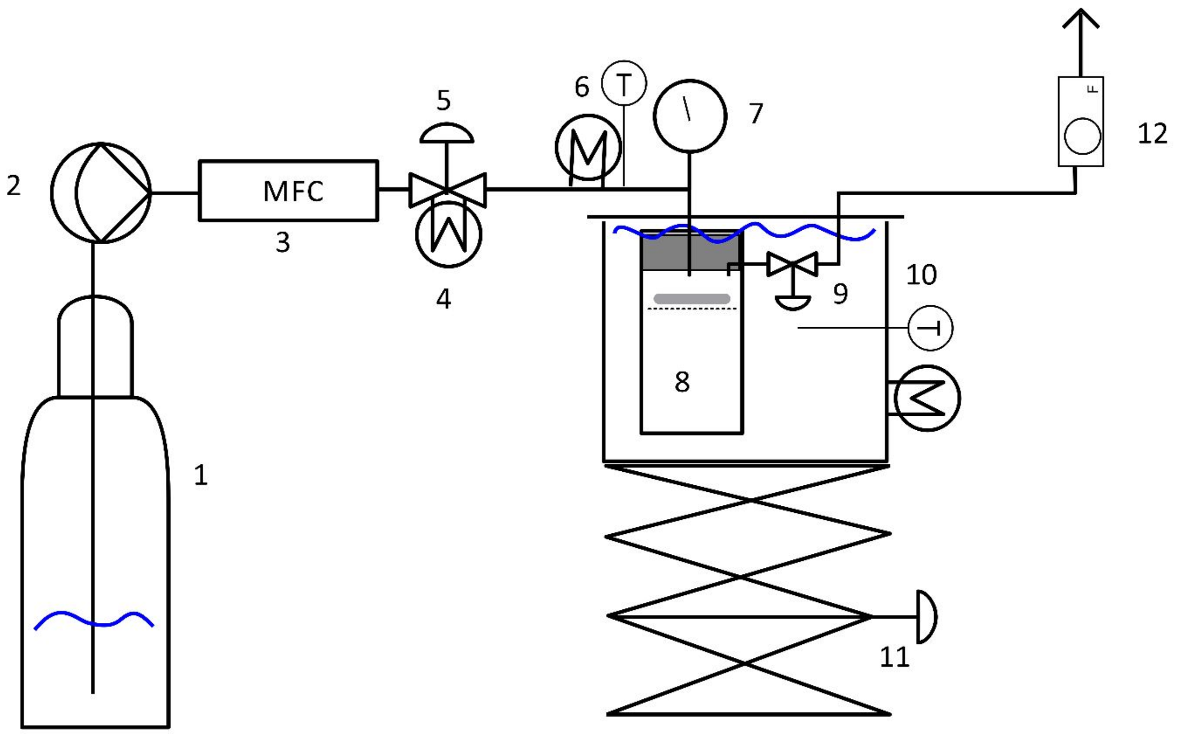

Figure S1. Schematic representation of the experimental setup (P&ID) for supercritical CO2 drying. 1. CO2 dip-tube bottle; 2. cooled high-pressure pump; 3. mass flow meter; 4. heater; 5. back pressure regulator; 6. pipe heating with thermocouple; 7. pressure gauge; 8. pressure separator with sample on stainless steel mesh; 9. proportional relief valve; 10. heating bath with thermocouples; 11. laboratory lift; 12. float-type flow meter.

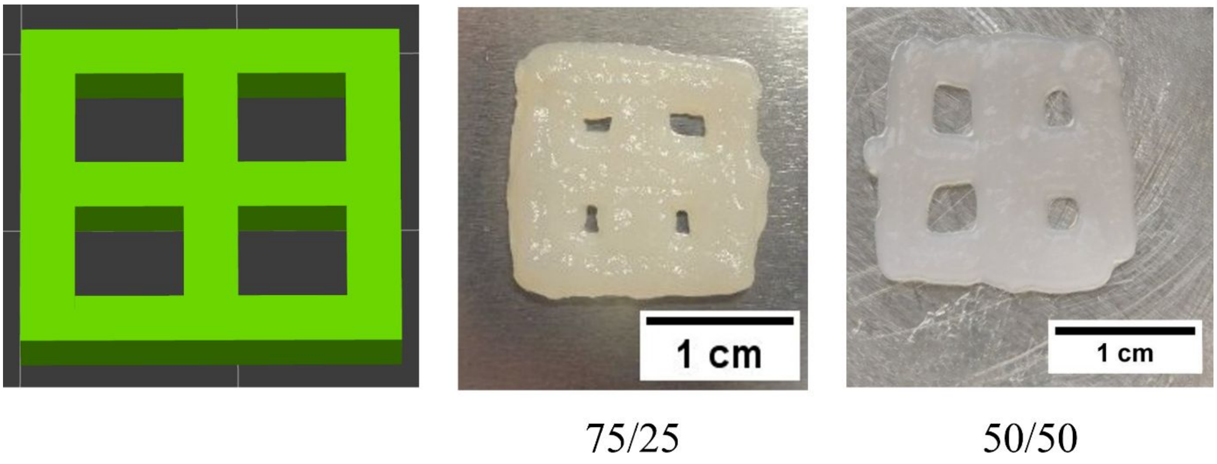

Figure S2. Left: 3D grid model with 17.5 x 17.5 x 20 mm dimension; Right: images of printed grids with the associated ratio below.

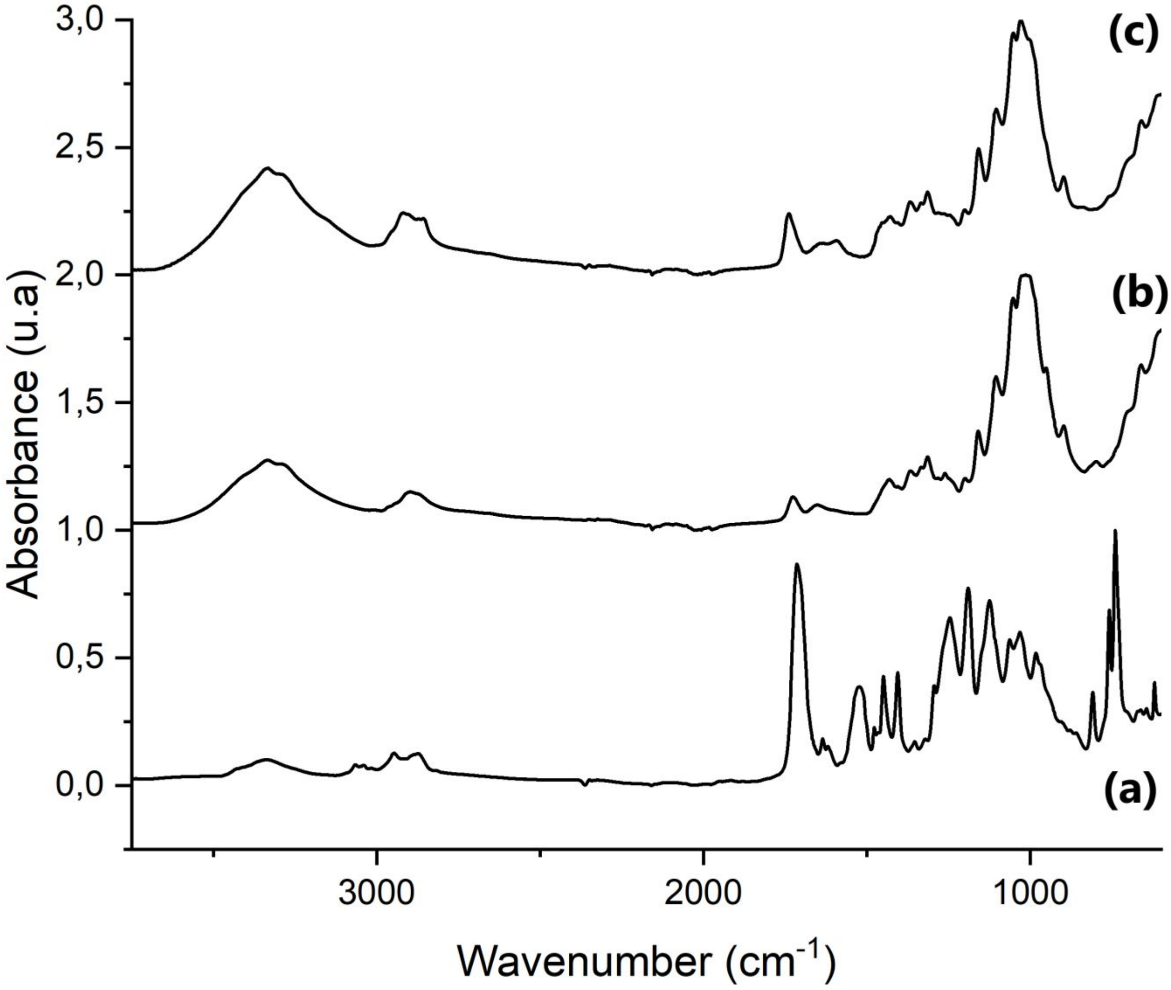

Figure S3: ATR-FTIR spectrum of pristine (a) Fmoc-AEEA, (b) CNF-MI, and (c) CNF-g-Fmoc

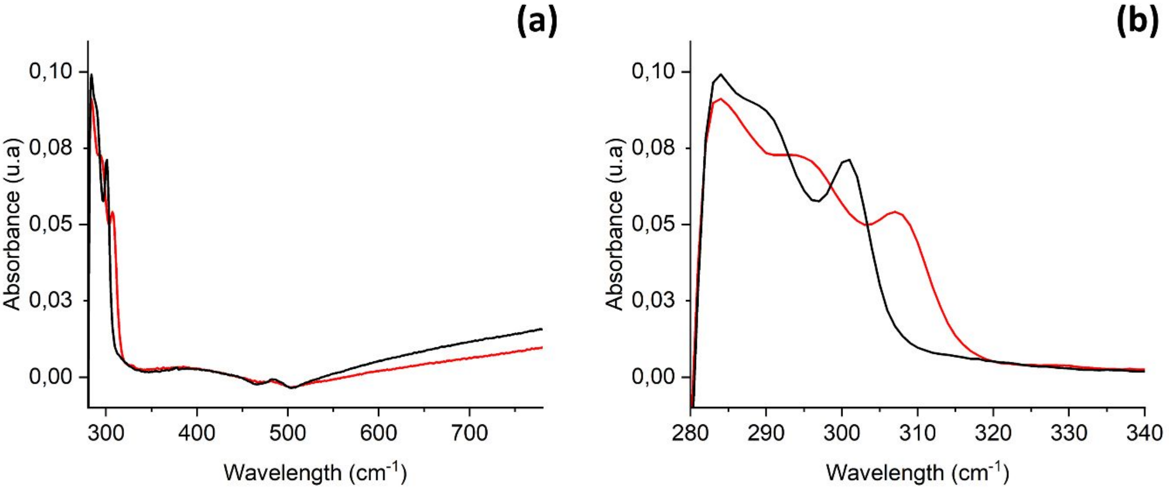

Figure S4: UV–Vis spectra of Fmoc-AEEA (C = 0.003248 M) in black, and CNF-g-Fmoc (C = 0.2 M) in red; (a) spectrum with wavenumber range from 280-800 cm^{-1}; (b) zoomed spectrum with wavenumber range from 280-340 nm^{-1}

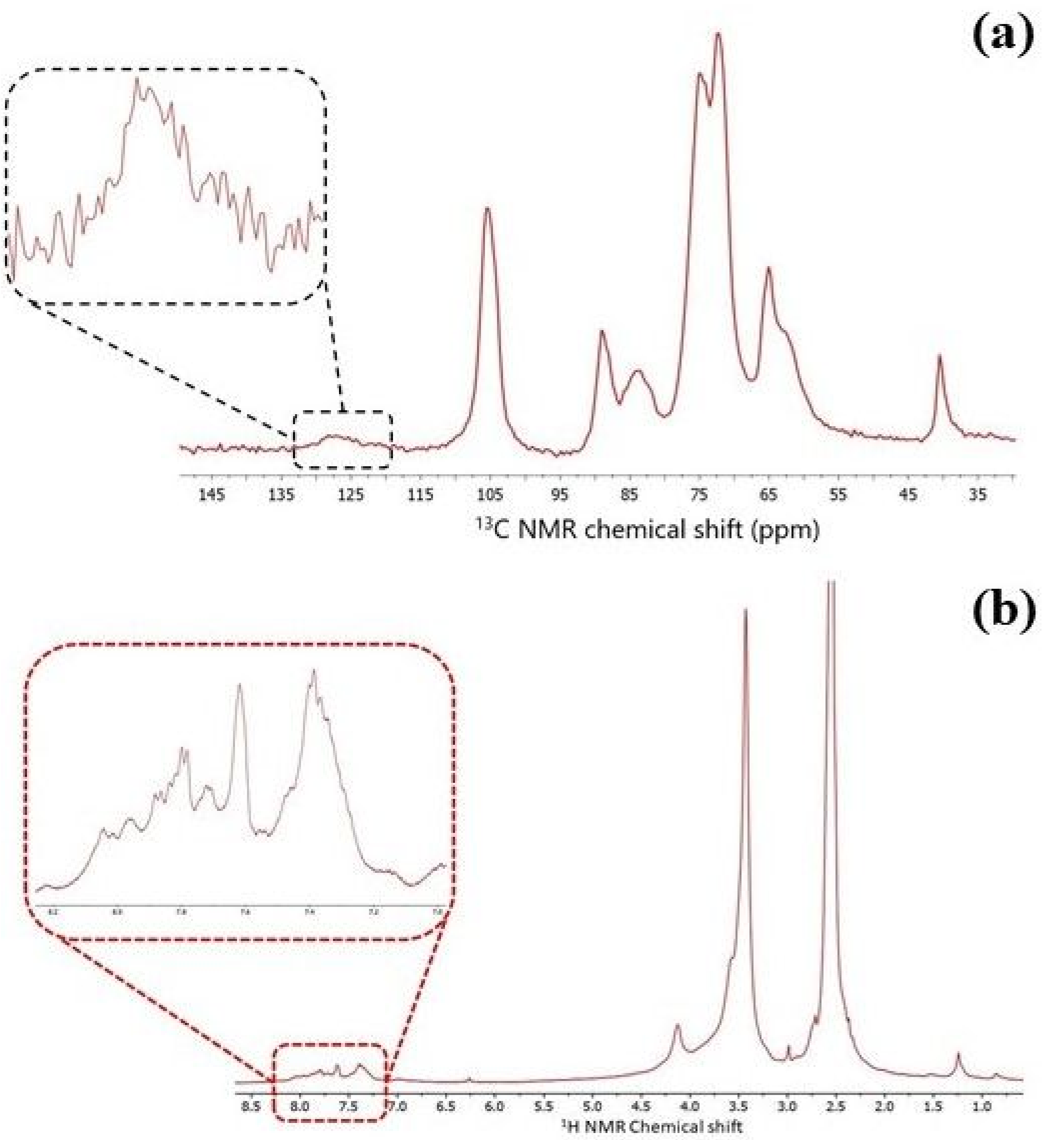

Figure S5. (a) Solid-state CP-MAS ^{13}C NMR spectra CNF-g-Fmoc; (b) ^{1}H HR-MAS NMR spectrum of CNF-g-Fmoc sample in DMSO-d_6

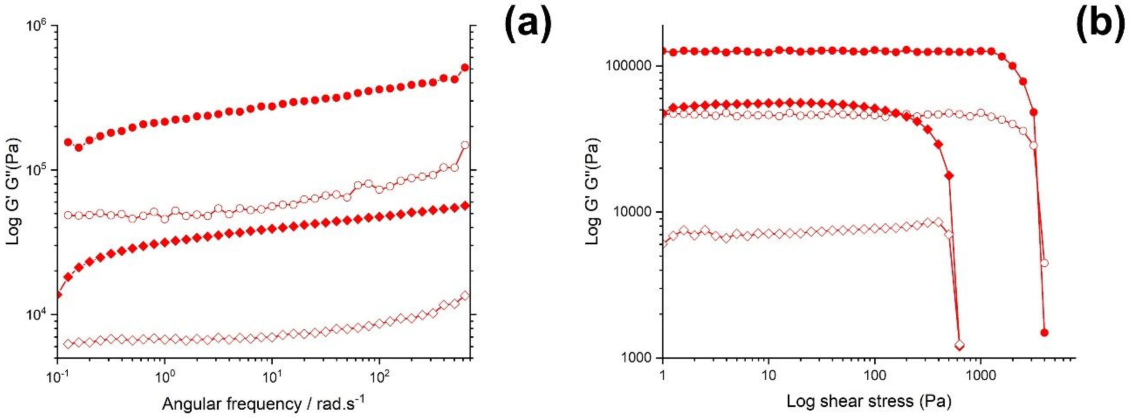

Figure S6: (a) Viscoelastic properties data and ddynamic modulus under increasing stress to determine yield stress (b) of *u*CNF/Fmoc-$FF_{3.89}$ before (○) and after mixing (◊); Full symbols for G' and hallow ones for G''.
